# Crystallization Mechanisms and Optical Properties of Yb^3+^-Containing Glasses and Glass-Ceramics: A Brief Review

**DOI:** 10.3390/ma19163476

**Published:** 2026-08-17

**Authors:** Xuebin Qiao, Xifeng Yang, Zihan Qiao, Taiju Tsuboi

**Affiliations:** 1 College of Electronic and Information Engineering, Suzhou University of Technology, Changshu 215500, China; 2 Jiangsu Key Laboratory of Advanced Laser Materials and Devices, School of Physics and Electronic Engineering, Jiangsu Normal University, Xuzhou 221116, China; 3 Department of Chemistry and Materials Science, Xi’an Jiaotong-Liverpool University, Suzhou 215000, China; 4 Faculty of Polymer Science and Engineering, Zhejiang University, Hangzhou 310027, China; 5 Faculty of Information Science and Engineering, Kyoto Sangyo University, Kamigamo, Kita-Ku, Kyoto 603-8555, Japan

**Keywords:** Yb^3+^-containing glass-ceramics, crystallization kinetics, nucleation and growth, rare-earth partitioning, upconversion, transparent photonic materials

## Abstract

Yb^3+^-containing glasses and glass-ceramics are attractive photonic materials because Yb^3+^ can act simultaneously as a near-infrared absorber, an energy-transfer sensitizer, a luminescent center, and a composition-dependent modifier of glass structure and crystallization. This brief review focuses on crystallization from parent glasses to glass-ceramics and examines glass-network chemistry, local Yb^3+^ coordination, phase separation, viscosity, heating rate, treatment temperature, holding time control nucleation, crystal growth, phase selection, rare-earth partitioning, transparency, and optical performance. Representative oxyfluoride, phosphate, oxyapatite, borosilicate, and aluminosilicate systems are compared using thermal analysis, X-ray diffraction, electron microscopy, vibrational spectroscopy, and optical spectroscopy. The available data show that Yb_2_O_3_ or YbF_3_ does not have a universal effect on crystallization: low concentrations can promote fluoride-rich clustering or lower the apparent crystallization barrier, whereas higher concentrations can increase packing density, stabilize the residual glass, change the competitive phase assemblage, or suppress crystallization. Crystallization-enhanced luminescence is most consistently obtained when Yb^3+^ and the activator partition into low-phonon-energy nanocrystals while crystal size and refractive-index mismatch remain sufficiently small to preserve transparency. This review also identifies major reporting gaps, including limited quantification of crystalline fraction, partition coefficients, luminescence lifetime, quantum efficiency, and long-term thermal stability. Practical design guidelines and unresolved questions are proposed to support the rational development of transparent Yb^3+^-containing glass-ceramics for lasers, sensing, optical amplification, and related photonic applications.

## 1. Introduction

### 1.1. Materials with and Without Yb^3+^ Ions

Numerous studies have been undertaken on the formation of crystal phases and crystalline fractions in glasses, the development of competing microstructures during crystallization, and the influence of atomic-size mismatch and composition on glass-forming ability [1,2,3,4,5,6,7,8]. A recent study by Contreras Jaimes and co-workers [9] examined bioactive glasses in the SiO_2_–P_2_O_5_–CaO–CaF_2_ quaternary system, focusing on how the orthophosphate-to-silicate ratio dictates phase development. Using a combination of XRD, solid-state NMR, and high-resolution imaging, they showed that phosphate-free compositions yield xonotlite, wollastonite, and cuspidine, whereas fluorapatite emerges only when P_2_O_5_ exceeds 2.4 mol%. Notably, crystallization remained marginal at low phosphate levels but intensified substantially above 3 mol% P_2_O_5_. The phosphate-free glass exhibited volume crystallization with nearly constant activation energy, while the high-phosphate variant displayed mixed surface–volume behavior and a declining activation energy with progressive conversion. These findings underscore the necessity of linking chemical makeup, thermal response, crystallization pathway, crystal fraction, grain size, and microstructure in rational glass-ceramic design [2,3,4,5,6,7,8,10,11,12,13].

Compared to the amount of research on SiO_2_-P_2_O_5_-CaO-CaF_2_ bioactive glasses without Yb^3+^ ions, systematic studies remain limited for glasses containing Yb^3+^ compounds, including Yb^3+^ glasses in which Yb_2_O_3_ or YbF_3_ acts as a functional constituent, although many reports have successfully identified the phases formed after heat treatment. Most studies adopt fixed concentrations for the major glass constituents and vary only the Yb^3+^-containing component or rare-earth codopant. The Yb^3+^ ion possesses a (4f)^13^(5s)^2^(5p)^6^ electronic configuration, in which the 4f electrons are effectively screened by the filled 5s and 5p subshells. This shielding renders the 4f→4f transitions largely insensitive to the surrounding ligand field. The ground state is ^2^F_7/2_, and the sole excited multiplet ^2^F_5/2_ lies approximately 10,000 cm^−1^ higher in energy. Consequently, Yb^3+^-doped glasses and glass-ceramics display a characteristic absorption band centered near 1000 nm. Moreover, when Yb^3+^ ions are positioned in close proximity to one another or to other lanthanides (e.g., Eu^3+^), the materials exhibit cooperative luminescence and sensitized upconversion via inter-ionic energy exchange [14,15,16,17,18,19,20,21,22,23,24,25]. 

Upconversion—often termed anti-Stokes emission—denotes the generation of higher-energy (shorter-wavelength) photons upon excitation with lower-energy (longer-wavelength) radiation. This phenomenon is frequently observed in various Ln^3+^-doped compounds, including those containing Yb^3+^. The upconversion property underpins applications in bioanalysis, theranostics (a technical term expressing the integration of therapeutics and diagnostics), biological imaging, optical sensing, optogenetics, super-resolution microscopy, barcoding, fingerprint detection, NIR vision, photochemical manipulation, optical trapping, additive manufacturing, laser technology, and NIR-II imaging [21,22,23,24,25,26,27,28,29,30,31,32,33,34,35]. For example, regarding the application to theranostics, the following report is presented [35]. Xu et al. reviewed recent advances in lanthanide-doped upconversion nanoparticles (Ln-UCNPs) as theranostic probes, emphasizing that their anti-Stokes luminescence, near-infrared excitation, large tissue-penetration depth, low photodamage, and efficient light-conversion capability make them attractive for biomedical diagnosis and treatment [35]. In particular, the higher-energy visible or ultraviolet light produced by Ln-UCNPs from NIR irradiation can be harnessed to trigger photosensitizers and photothermal agents, facilitating photodynamic therapy as well as photothermal therapy. Through surface modification and coupling with functional molecules, they can also act as drug-delivery vehicles, optical imaging probes, biosensors, and multimodal platforms for computed tomography, magnetic resonance imaging, fluorescence imaging, and image-guided therapy. These theranostic examples clearly indicate that Yb^3+^-sensitized upconversion materials are not only important for understanding energy-transfer and luminescence mechanisms but also provide a versatile materials basis for integrated diagnosis, treatment, and biomedical monitoring.

### 1.2. Microstructures of Glass, Glass-Ceramics, and Ceramics

**Figure 1:**
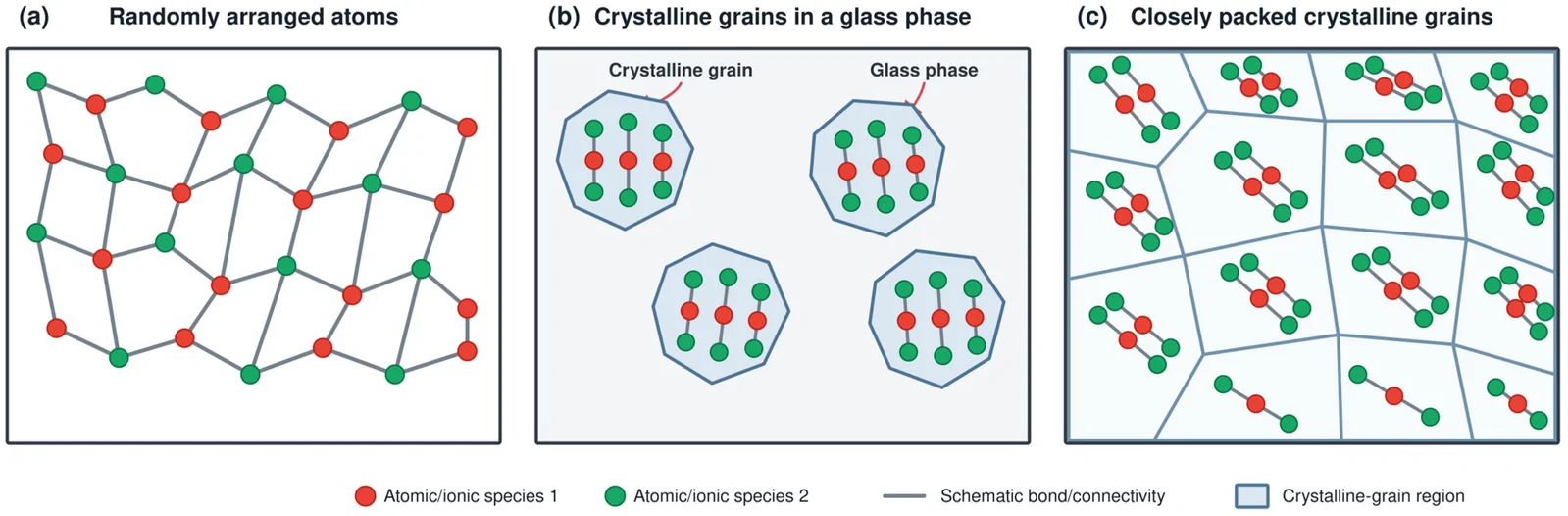
Schematic comparison of atomic arrangements in (**a**) an amorphous glass with randomly arranged atoms, (**b**) a glass-ceramic containing crystalline grains dispersed in a residual glass phase, and (**c**) a polycrystalline material composed of closely packed crystalline grains. The red and green spheres schematically distinguish two different atomic/ionic species and do not represent specific elements. The gray lines indicate local atomic connectivity, the blue outlines denote crystalline-grain boundaries, and the light-blue regions represent crystalline-grain regions. Reproduced with permission [36]. Copyright 2020, MDPI.

Glass-ceramics can be formed from parent glasses via controlled nucleation and crystallization, both of which are induced by appropriate heat-treatment schedules. Depending on the thermal schedule and composition, the microstructure evolves from an amorphous glass to a glass-ceramic and, ultimately, to a more fully crystallized ceramic material [2,3,4,5]. A schematic illustration of the nucleation and crystallization processes in glass during thermal treatment is shown in Figure 1 [36]. During glass-ceramic formation, the crystalline phases, crystal sizes, residual glass composition, and spatial distribution of crystals are strongly governed by the parent-glass composition, nucleating agents, heating rate, holding temperature, and holding time [2,3,4,5,6,7,8].

A glass is an amorphous solid lacking translational periodicity in its atomic arrangement (Figure 1a). A glass-ceramic is a two-phase material composed of one or more crystalline species dispersed in an amorphous residual phase (Figure 1b). Within this composite, the crystalline constituents—specifically their size, morphology, orientation, and abundance—dictate the resulting optical, thermal, mechanical, and chemical responses [2,3,6,7]. A fully crystalline ceramic (Figure 1c), by contrast, is essentially an assembly of crystal grains, with only a negligible glassy fraction at the grain boundaries. The ordered unit cells in each grain provide sharp XRD reflections, whereas the random orientation of grains produces a polycrystalline diffraction pattern. 

### 1.3. Scope and Literature-Selection Strategy

This review draws on a focused selection of peer-reviewed studies published between 1959 and 2026. Web of Science, Scopus, and Google Scholar were searched using combinations of keywords including “ytterbium glass-ceramic crystallization,” “Yb_2_O_3_ glass crystallization,” “YbF_3_ oxyfluoride glass-ceramic,” “rare-earth partitioning,” “crystallization kinetics,” “Avrami,” “DSC,” “XRD,” “TEM,” “Raman,” “XANES,” “EXAFS,” “upconversion,” and other composition-specific terms. Primary studies were considered eligible if they reported at least two of the following: parent-glass composition, thermal treatment schedule, crystallization temperature or kinetic parameters, identified crystalline phase, microstructure or grain size, evidence of rare-earth partitioning, transparency, or luminescence. Studies devoted solely to single-crystal growth or ceramic-powder processing were excluded from the core analysis unless they provided essential measurement data on Yb^3+^ energy levels or phase identification. This selection strategy ensures that this review remains focused on the glass-to-glass-ceramic transformation rather than on general ceramic processing. The final body of literature was further enriched with mechanistic investigations into phase separation, interface-controlled growth, optical refrigeration, Yb-mediated phase selection, and optical thermometry [37,38,39,40,41,42,43,44,45,46,47,48,49,50,51,52,53,54,55,56,57,58,59,60,61].

This review is organized around composition–processing–structure–property relationships. Table 1 integrates crystallization conditions, kinetic and structural evidence, Yb^3+^ partitioning, optical performance, transparency, thermal stability, and principal reporting gaps, while distinguishing directly measured quantities from interpretations and explicitly marking data that were not reported. This is important because enhanced emission alone does not prove that Yb^3+^ has entered a crystalline phase, and the absence of sharp XRD peaks does not exclude a small fraction of very small or weakly scattering nanocrystals.

## 2. Mechanistic Framework for Crystallization in Yb^3+^-Containing Glasses

Crystallization emerges from the interplay between thermodynamic driving force and atomic/ionic mobility. Upon cooling from the melt, the chemical-potential disparity between the supercooled liquid and a potential crystalline phase widens, yet the accompanying viscosity increase progressively hinders structural reconfiguration. For nucleation to proceed, the volumetric free-energy reduction must compensate for the energetic penalty associated with forming a crystal–glass interface. Growth, in turn, becomes kinetically accessible only when the relevant species can migrate through the adjacent glassy medium. As a consequence, the temperature at which nucleation peaks is typically lower than that for maximum growth—a relationship that underpins the common practice of two-step thermal treatments. In multicomponent glasses, phenomena such as liquid–liquid demixing, the clustering of network-modifying cations, or fluoride-enriched zone formation can diminish the effective interface energy and serve as heterogeneous nucleation promoters [1,2,3,4,5,6,7,8].

Yb^3+^ can influence each of these factors. Owing to its high field strength and specific coordination demands, Yb^3+^ may depolymerize one structural network while reinforcing another, alter the abundance of non-bridging oxygen species, modulate fluoride or phosphate bonding environments, and affect viscous flow. When Yb^3+^ partitions into a local region that compositionally prefigures the forthcoming crystalline phase, nucleation tends to be accelerated. Conversely, if Yb^3+^ enhances configurational entropy or stabilizes the residual amorphous framework, crystallization may be slower. The net outcome is system-dependent and must be determined within a well-defined compositional series; generalizations across silicate, phosphate, borate, and oxyfluoride systems are not warranted.

Heating rate introduces additional complexity in comparative studies. Under faster DSC or DTA ramps, the crystallization exotherm shifts upward in temperature because the system dwells for shorter durations within the window where nuclei and crystals can develop. Thus, the peak temperature is not a fixed intrinsic property. Furthermore, a given exotherm may reflect different underlying events, nucleation combined with growth, the growth of pre-existing nuclei, the concurrent formation of multiple phases, or surface-dominated crystallization. Reliable interpretation requires data collected over a range of heating rates, complemented by the phase-resolved characterization of specimens quenched before, at, and beyond each thermal signature [10,11,12,13].

### 2.1. Kinetic Analysis and Its Limits

The Kissinger method is widely employed to extract an apparent activation energy *E_a_* from crystallization peaks recorded at various linear heating rates *β*. The relationship is given by

ln(*β*/*T_p_*^2^) = −*E_a_*/(*RT_p_*) + *C* (1)

where *T_p_* denotes the exotherm maximum, *R* is the gas constant, and *C* is a constant [10]. Alternative Ozawa-type treatments use a different functional form of *β* and can be applied either to peak shifts or to a specified fractional conversion [11]. The term “apparent” is used advisedly, as the derived *E_a_* may reflect contributions from transport processes, interfacial reactions, and evolving phase compositions simultaneously. A reduction in *E_a_* upon Yb addition can be interpreted as enhanced transformability only when the compared specimens follow identical reaction routes and yield the same crystalline product.

The Johnson–Mehl–Avrami–Kolmogorov framework relates the transformed fraction α to time t through

ln[−ln(1 − *α*)] = *n* ln(*t*) + *C*′ (2)

yielding an Avrami exponent *n* under stipulated assumptions. In glass research, *n* is commonly estimated from non-isothermal variants of this formalism [12,13]. Although values of *n* near unity are often linked to surface-dominated growth or one-dimensional/diffusion-controlled mechanisms, morphology, pre-existing nuclei, and time-dependent nucleation can generate comparable fitted numbers. Hence, inferring a specific mechanism solely from the Avrami exponent is unjustified. Such assignments must be corroborated by microscopy observations, particle-size dependence, and the progression of XRD patterns.

Viscosity measurements offer a crucial link between thermal signatures and the underlying structural behavior. A Vogel–Fulcher–Tammann parametrization may assist in comparing the temperature sensitivity of mobility, though extrapolation beyond the measured range carries considerable uncertainty [78]. Phase-change materials further demonstrate that crystallization pathways can alternate between glass-controlled and undercooled-liquid-controlled regimes [79]. For Yb^3+^-containing glass-ceramics, the most informative design strategy integrates viscosity, nucleation rate, growth kinetics, and phase identification rather than depending on a single thermal stability criterion such as ΔT = T_x_ − T_g_.

**Figure 2:**
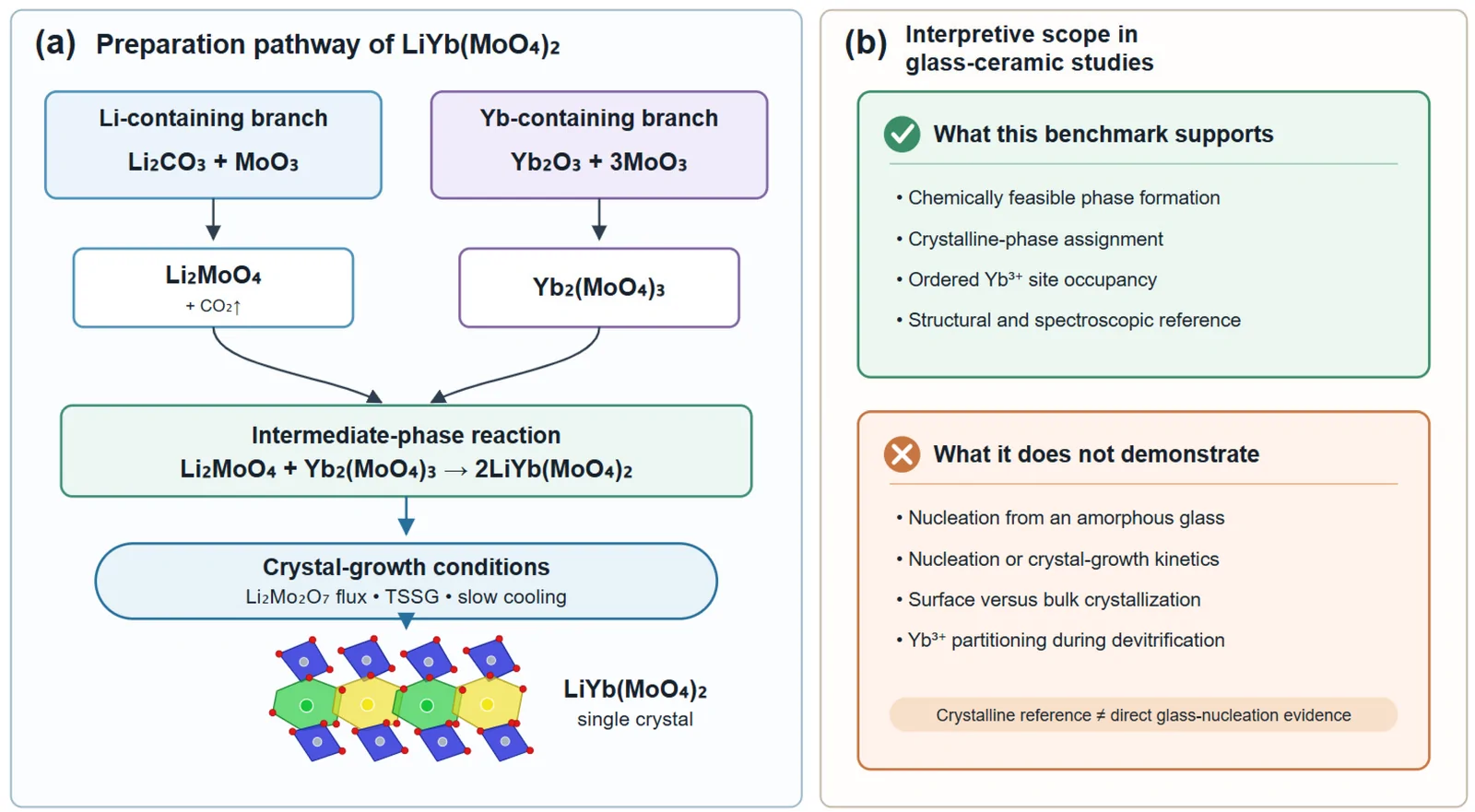
Author-created schematic illustrating the preparation pathway and interpretive scope of LiYb(MoO_4_)_2_ as a crystalline benchmark. (**a**) Formation of Li_2_MoO_4_ and Yb_2_(MoO_4_)_3_ intermediates from oxide/carbonate precursors, followed by their reaction to produce LiYb(MoO_4_)_2_ and subsequent crystal growth using a Li_2_Mo_2_O_7_ flux, top-seeded solution growth (TSSG), and slow cooling. A simplified polyhedral representation of the LiYb(MoO_4_)_2_ crystal structure is shown at the bottom. (**b**) Information that this crystalline benchmark can and cannot provide for interpreting glass-ceramic crystallization. The synthesis pathway was constructed by the authors using information reported in Ref. [80], and all graphical elements were created by the authors.

The crystalline LiYb(MoO_4_)_2_ system is retained only as a limited benchmark for phase chemistry, site occupancy, and Yb^3+^ spectroscopy. Because it is prepared by solution-mediated crystal growth or powder-based solid-state synthesis, it does not provide direct evidence for nucleation from an amorphous glass. Figure 2 summarizes its chemically feasible preparation pathway and explicitly defines the conclusions that can and cannot be drawn from this crystalline benchmark [80].

### 2.2. LiYb(MoO_4_)_2_ as a Structural and Spectroscopic Benchmark

LiYb(MoO_4_)_2_ is a tetragonal double molybdate in which Yb^3+^ occupies a stoichiometric lattice position rather than being present as a dilute dopant. This compound has drawn interest as a potential laser host, owing to the relatively uncomplicated electronic configuration of Yb^3+^, characterized by the ^2^F_7/2_ ground multiplet and the ^2^F_5/2_ excited multiplet. Its absorption features lie predominantly in the 0.9–1.0 μm range, with near-infrared emission centered near 1.0 μm [14,15,16,17,80,81]. The well-defined periodicity of this crystalline lattice serves as a useful reference for validating energy-level assignments and for detecting spectral modifications that arise from rare-earth ions occupying ordered sites. It does not, however, offer direct insight into the influence of Yb^3+^ on nucleation energetics or crystal-growth kinetics within an amorphous matrix.

Because LiYb(MoO_4_)_2_ undergoes incongruent melting at approximately 912 °C, Volkov and co-workers [80] adopted a flux-based approach using Li_2_Mo_2_O_7_, combined with top-seeded solution growth, rather than conventional Czochralski pulling. As summarized in Figure 2a, Li_2_MoO_4_ and Yb_2_(MoO_4_)_3_ were first prepared as intermediate compounds from Li_2_CO_3_, MoO_3_, and Yb_2_O_3_. These intermediates were subsequently reacted to form LiYb(MoO_4_)_2_, which was followed by crystal growth using a Li_2_Mo_2_O_7_ flux and slow cooling. Crystals approximately 12 mm in size were obtained by initiating growth just below the decomposition temperature. The simplified polyhedral representation at the bottom of Figure 2a illustrates the ordered crystalline structure of LiYb(MoO_4_)_2_. This route represents solution-mediated single-crystal growth rather than glass devitrification and is included only to establish a chemically feasible formation pathway and an ordered structural reference for Yb^3+^.

The oxidation-state balance of the precursor chemistry also requires clarification. In LiYb(MoO_4_)_2_, Mo is present as Mo^6+^ in the [MoO_4_]^2−^ group; therefore, MoO_3_ is the direct oxidation-state-matched precursor used in the preparation route summarized in Figure 2a [80]. If MoO_2_ is employed in an alternative preparation route, the oxidation of Mo^4+^ to Mo^6+^ during heat treatment must occur and should be explicitly considered when interpreting the phase-formation mechanism. More generally, crystalline phases assigned in heat-treated glasses should be chemically compatible with the original batch composition and independently verified by phase-sensitive characterization rather than inferred from optical spectra alone.

Figure 2b clarifies the interpretive scope of crystalline LiYb(MoO_4_)_2_ in the present review. This crystalline benchmark provides useful reference information for assessing chemically feasible phase formation, crystalline-phase assignments, ordered Yb^3+^ site occupancy, and structural and spectroscopic characteristics. However, it does not directly demonstrate nucleation from an amorphous glass, nucleation or crystal-growth kinetics, surface versus bulk crystallization, or Yb^3+^ partitioning during glass devitrification.

### 2.3. Powder-Derived Reference Materials: Relevance and Limitations

**Figure 3:**
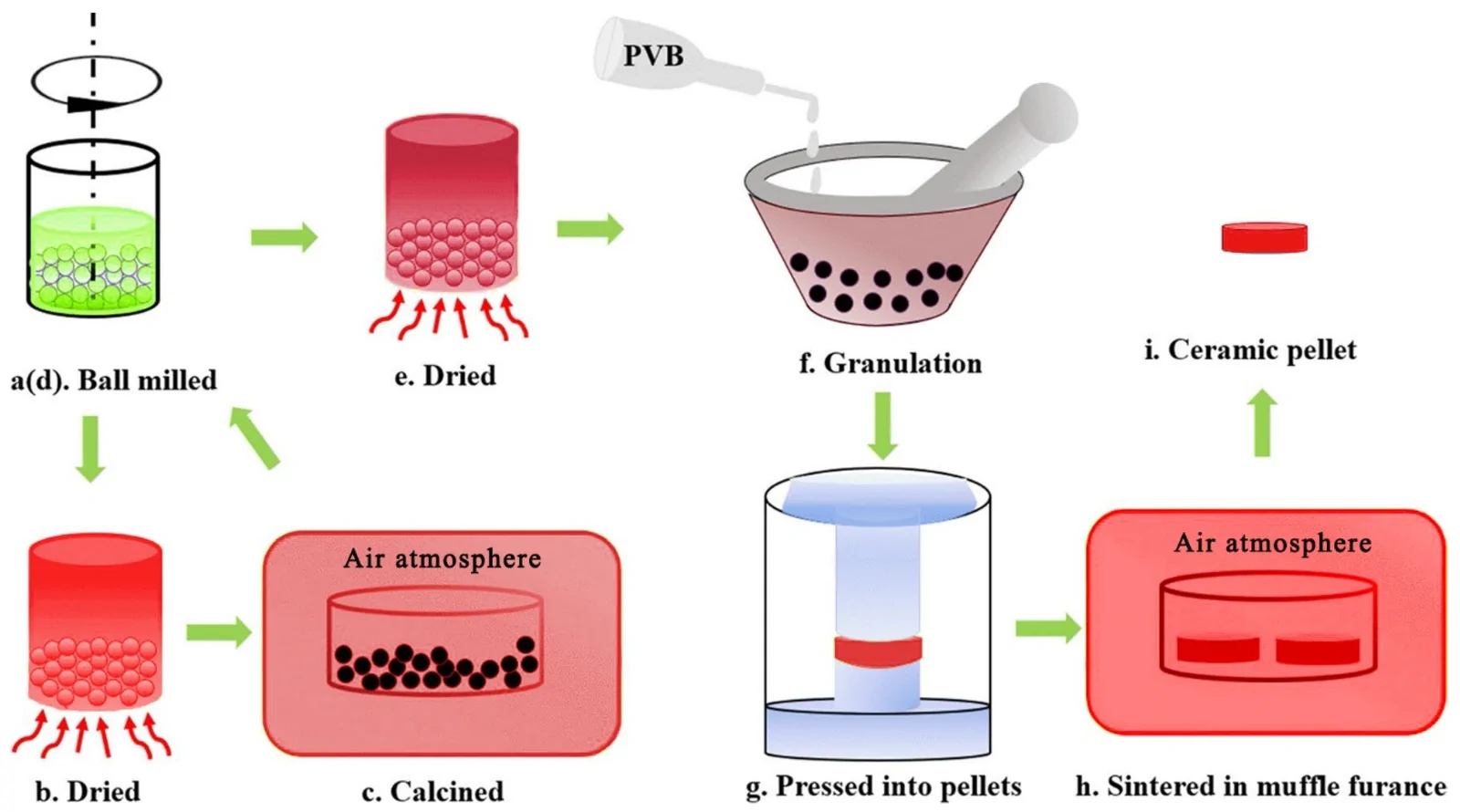
Schematic condensed preparation route for Ln^3+^-substituted LiYb(MoO_4_)_2_ crystals, where Ln denotes lanthanides such as Er and Eu. Green arrows indicate the processing sequence; the green region denotes the liquid milling medium; red-shaded regions indicate drying or heat treatment in air; red and black spheres represent powder particles and granules, respectively; the blue region represents uniaxial pressing; and PVB denotes the poly(vinyl butyral) binder used during granulation.

Powder-derived LiYb_1−x_Ln_x_(MoO_4_)_2_ has also been prepared by milling stoichiometric Li_2_CO_3_, MoO_3_, Yb_2_O_3_, and Ln_2_O_3_, followed by staged calcination and sintering in air [82]. In this route, precursor purity, particle size, mixing, temperature, and atmosphere determine the phase purity of the final crystalline material [83,84]. These processing variables are relevant to solid-state synthesis but do not directly explain nucleation within a glass. Accordingly, general descriptions of press types, green-body mechanics, calcination, and ceramic sintering are not considered further in this review. Such a synthesis method is illustrated in Figure 3. 

The above synthesis route was also used to prepare LiYb_1-x_Er_x_ (MoO_4_)_2_ (0.01 ≤ x ≤ 1) crystals by the present authors [82], where the starting materials of Er_2_O_3_, Yb_2_O_3_, and 99.9% Li_2_CO_3_ and MoO_2_ were weighted by an appropriate stoichiometric ratio, thoroughly mixed in an agate mortar, and transferred to a corundum crucible to sinter. The mixture powder was first pre-fired at 300 °C for 5 h and then at 500 °C for 5 h and finally heated at 800 °C for 8 h in air for preparation of the final products of the phosphors. During every step of the heat treatment, grinding and homogeneous mixing were employed. 

The above powder-derived material method provides excellent crystallization formation, which is confirmed by the crystalline phase diagram measurement. XRD can verify the final crystalline phase, while microscopy and photoluminescence characterize morphology and optical response. For a glass-ceramic, however, these measurements alone cannot determine when nuclei first formed, whether crystallization occurred at the surface or throughout the volume, what fraction of the glass crystallized, or what proportion of Yb^3+^ entered the crystalline phase. The crystalline benchmark should therefore support phase and spectroscopic assignments but should not substitute for kinetic measurements or the direct analysis of Yb^3+^ partitioning.

### 2.4. Substitutional Chemistry and Its Relevance to Glass-Ceramics

**Figure 4:**
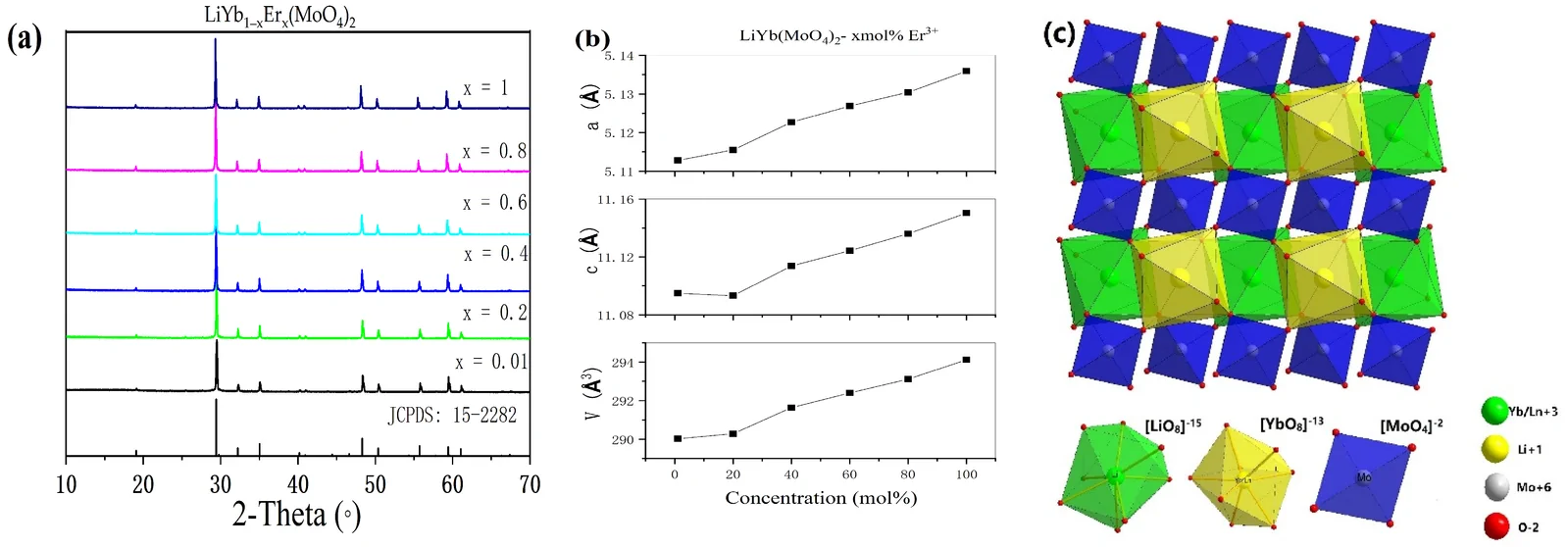
(**a**) XRD patterns of LiYb_1–x_Er_x_(MoO_4_)_2_ (x = 0.01–1.00), with the reference pattern; (**b**) variation in the unit-cell parameters as a function of x. Panels (**a**,**b**) are reproduced with permission from Ref. [82]. Copyright 2024, Elsevier B.V. (**c**) Schematic representation of the crystal structure of LiYb(MoO_4_)_2_, created by the authors. The structure is illustrated by taking into account the oxygen coordination geometries of the metal sites occupied by Yb, Mo, and Li ions.

Substitution of Ln^3+^ for Yb^3+^ in LiYb_1−x_Ln_x_(MoO_4_)_2_ is facilitated by their identical charge and comparable coordination-dependent ionic radii. In LiYb_1−x_Er_x_(MoO_4_)_2_, the tetragonal structure is retained over the investigated composition range, and the lattice parameters vary approximately with Er^3+^ content, consistent with the substitution of Er^3+^ at the Yb^3+^ site [82,85,86]. Figure 4 summarizes the corresponding XRD patterns and composition-dependent unit-cell parameters.

This substitutional behavior is relevant to glass-ceramics because crystal-field splitting, emission line shapes, and lifetime components may indicate whether Yb^3+^ or a codoped activator has entered an ordered crystalline environment. Nevertheless, optical evidence remains indirect: similar spectra may arise from different local coordination environments, and an approximately linear change in lattice parameters does not quantify the fraction of rare-earth ions remaining in the residual glass. Direct or quantitative methods—including EXAFS/XANES, site-selective spectroscopy, spatially resolved elemental analysis, and multicomponent lifetime analysis—are required to determine rare-earth partitioning reliably. The LiYb(MoO_4_)_2_ system is therefore used only as a structural and spectroscopic reference and is not generalized as a model for the thermodynamics or kinetics of glass crystallization.

## 3. Optical Processes Relevant to Yb^3+^-Containing Glass-Ceramics

### 3.1. Upconverted Emission of Yb^3+^-Doped Crystals

#### 3.1.1. Cooperative Luminescence (CL)

A broad emission feature centered at ~497 nm (20,120 cm^−1^) was first observed in YbPO_4_ by Nakazawa and Shionoya [87] in 1970. This signal appeared under near-infrared pumping near 1000 nm at ambient temperature, with the band spanning from ~480 nm to ~520 nm on its high- and low-energy sides, respectively. The emission energy is approximately twice the excitation energy, and its intensity increases nearly quadratically with pump power. This behavior was assigned to cooperative luminescence (CL), which originates from a pair of neighboring Yb^3+^ ions through a two-photon upconversion process [18,19,87]. This cooperative process follows the reaction scheme:

Yb^3+^(^2^F_5/2_) + Yb^3+^(^2^F_5/2_) → Yb^3+^(^2^F_7/2_) + Yb^3+^(^2^F_7/2_) + CL (3)

This mechanism is thought to proceed through an intermediate [^2^F_5/2_ ^2^F_5/2_] state, which arises from electronic coupling between two neighboring Yb^3+^ ions—one in the ^2^F_5/2_ excited level and the other also in the ^2^F_5/2_ state.

**Figure 5:**
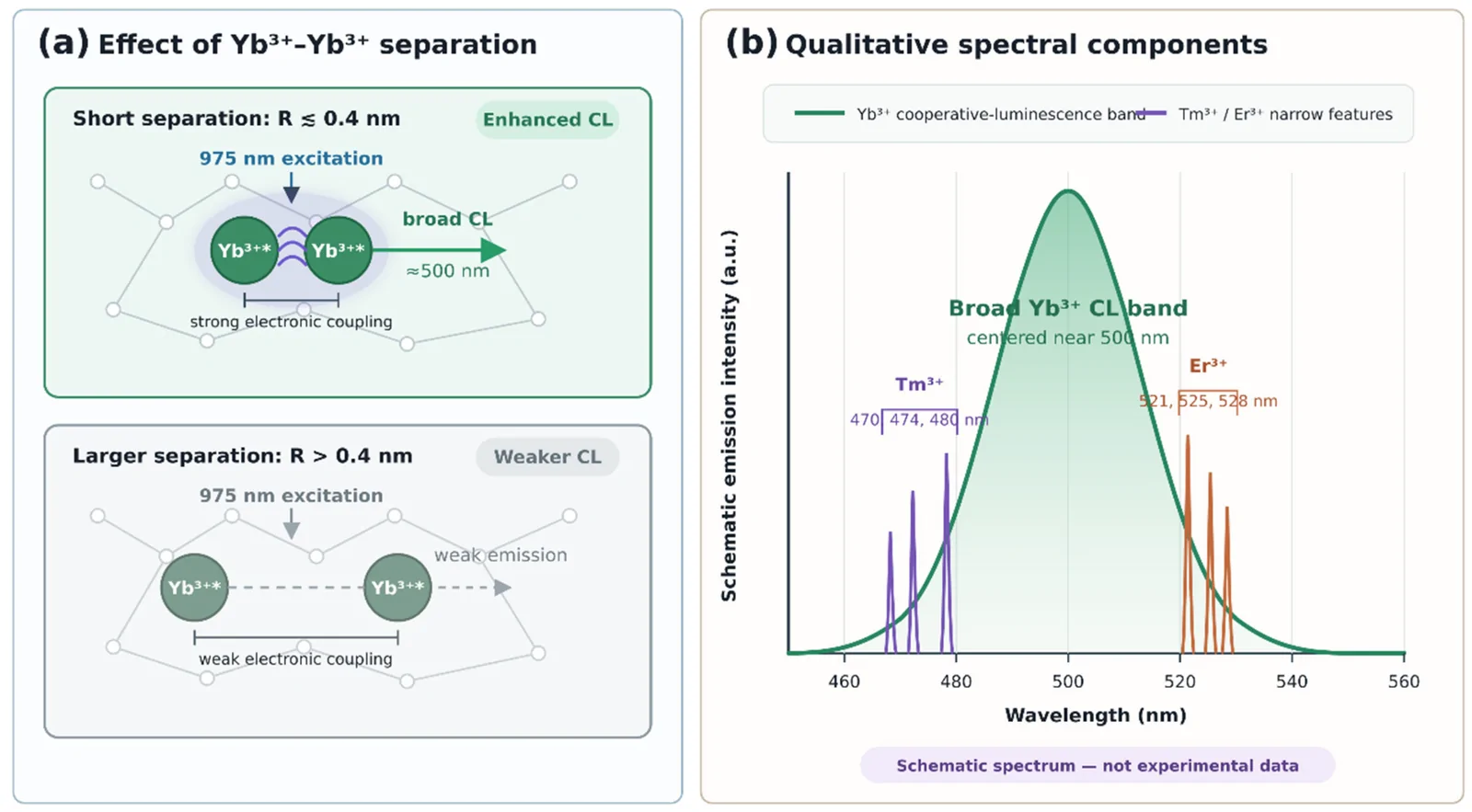
Author-created schematic illustrating the influence of Yb^3+^–Yb^3+^ separation and rare-earth impurities on cooperative luminescence. (**a**) Under 975 nm near-infrared excitation, a short Yb^3+^–Yb^3+^ separation (R ≲ 0.4 nm) promotes strong electronic coupling between neighboring excited Yb^3+^ ions and enhances cooperative luminescence near 500 nm, whereas a larger separation weakens the electronic coupling and suppresses the cooperative process. The asterisk (*) in “Yb^3+^” denotes an Yb^3+^ ion in its excited state. (**b**) Schematic spectral representation of the broad Yb^3+^ cooperative-luminescence band centered near 500 nm, with representative narrow Tm^3+^- and Er^3+^-related emission features superimposed. The spectrum is provided for conceptual illustration and does not represent digitized experimental data. The scientific concepts and representative wavelength assignments are based on Refs. [87,88], and all graphical elements were created by the authors.

Since its initial observation in YbPO_4_, a broad cooperative-luminescence band near 500 nm has been reported in numerous Yb^3+^-containing crystals, glasses, ceramics, and solutions [18,19,20,87,88]. In Yb^3+^-doped phosphate glasses, the probability of cooperative luminescence increases as the average Yb^3+^–Yb^3+^ separation decreases, with pronounced cooperative emission reported when the separation is shorter than approximately 0.4 nm [88]. As schematically illustrated in Figure 5a, the shorter inter-ionic distance enhances electronic coupling between neighboring excited Yb^3+^ ions, whereas a larger separation suppresses the cooperative process. The resulting emission is characterized by a broad band centered near 500 nm. Narrow emission features associated with intentionally or unintentionally incorporated rare-earth ions may be superimposed on this band, as illustrated schematically in Figure 5b. Representative Tm^3+^-related emissions occur near 470, 474, and 480 nm, whereas Er^3+^-related emissions have been reported near 521, 525, and 528 nm [87,88].

**Figure 6:**
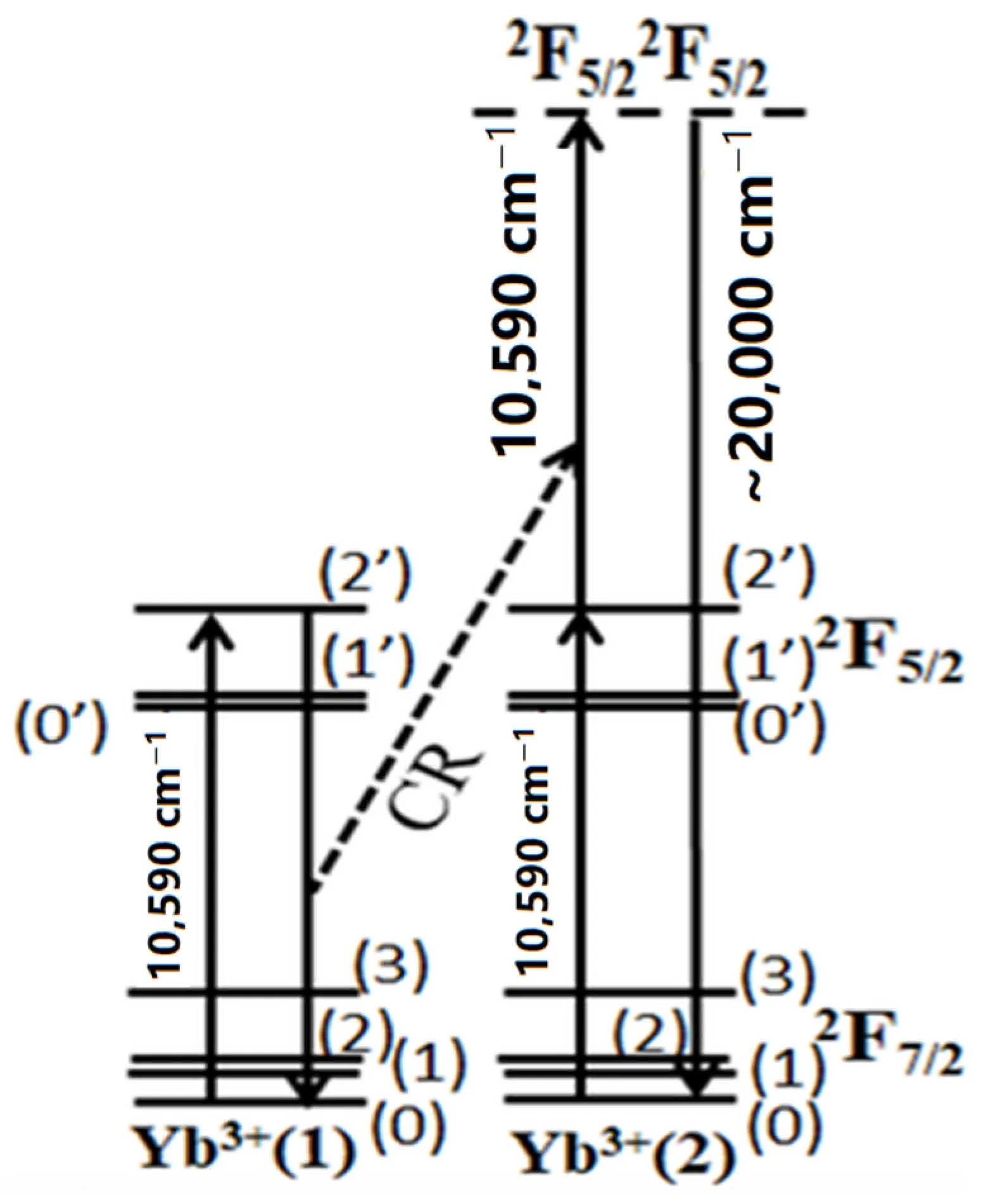
Schematic energy-level diagram illustrating cooperative luminescence (CL) from a Yb^3+^ dimer with a short Yb^3+^–Yb^3+^ separation. Under 944 nm excitation, cross-relaxation (CR) occurs between two neighboring Yb^3+^ ions, denoted Yb^3+^(1) and Yb^3+^(2), and CL is assumed to originate from Yb^3+^(2). Labels such as (1) denote crystal-field-split Stark substates of the ^2^F_7/2_ ground state, whereas labels such as (2′) denote substates of the ^2^F_5/2_ excited state. The corresponding energy gaps are labeled in cm^−1^.

A simplified model for the CL mechanism is shown in Figure 6 [18,19,87,88]. Two neighboring Yb^3+^ ions, Yb^3+^(1) and Yb^3+^(2), are simultaneously excited to the ^2^F_5/2_ state under pumping at 10,590 cm^−1^. The donor Yb^3+^(1) relaxes non-radiatively to the ground state and passes its energy to the adjacent Yb^3+^(2), which is already in the excited ^2^F_5/2_ state, promoting it to an intermediate Yb^3+^–Yb^3+^ dimer state denoted as [^2^F_5/2_ ^2^F_5/2_] at approximately 21,180 cm^−1^ (= 2 × 10,590 cm^−1^). This cross-relaxation-type energy-transfer process is followed by radiative emission from the dimer state, producing CL.

#### 3.1.2. Yb^3+^-Sensitized Rare-Earth Emission

**Figure 7:**
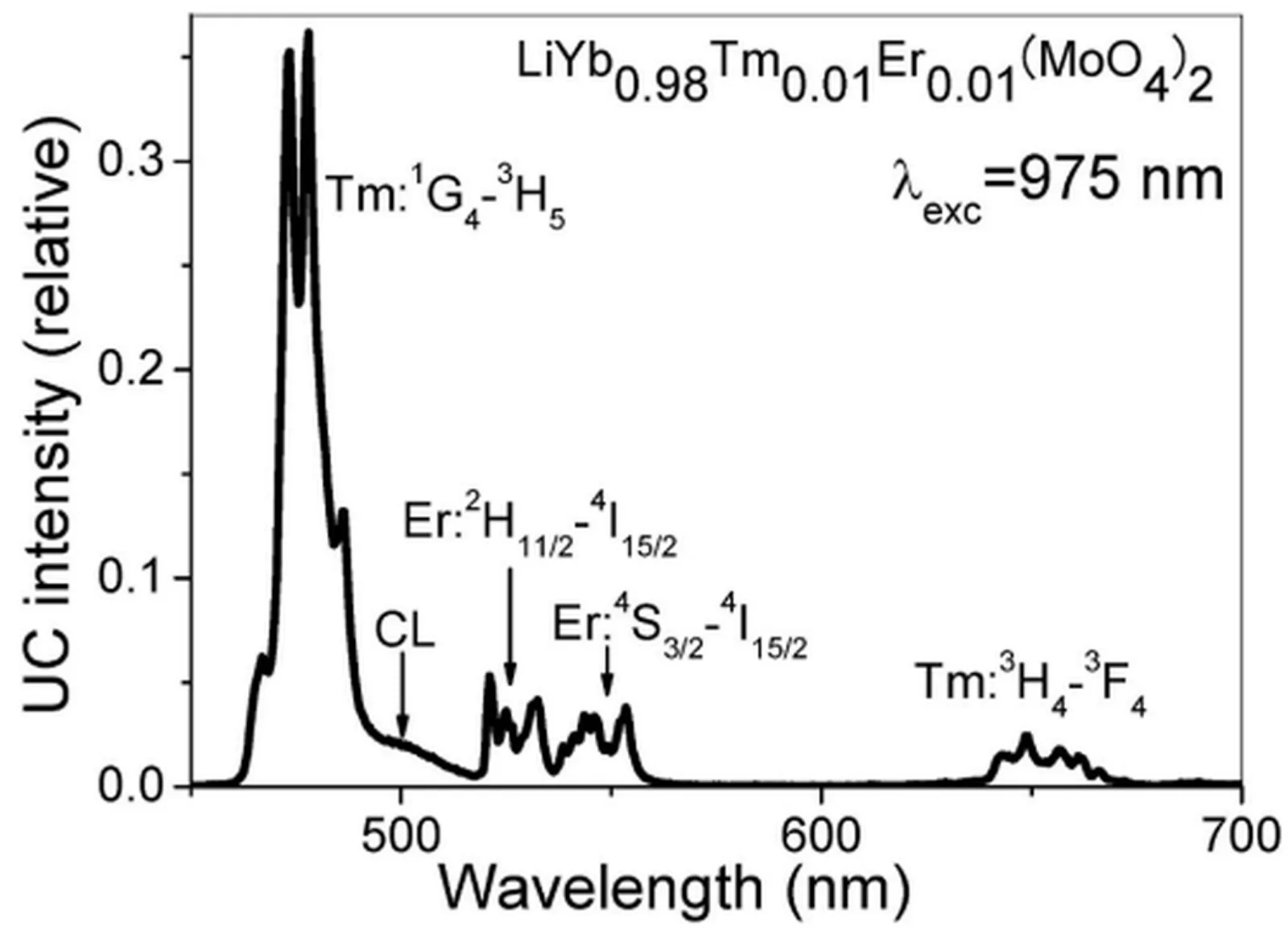
Upconversion spectrum of LiYb_0.98_Tm_0.01_Er_0.01_(MoO_4_)_2_ under 975 nm excitation, showing rare-earth emission superimposed on part of the broad Yb^3+^ cooperative-luminescence band. Reproduced with permission from Ref. [82]. Copyright 2024, Elsevier B.V.

In addition to the broad cooperative-luminescence band near 500 nm, narrow upconversion-emission features may appear when trivalent lanthanide ions such as Er^3+^, Ho^3+^, Tb^3+^, or Tm^3+^ are intentionally or unintentionally codoped into Yb^3+^-containing compounds, as schematically illustrated in Figure 7 [18,19,20,21,22,23,24,25,31,32,33,34,63,82,87,88]. Each upconversion emission typically exhibits a power-law relationship with the excitation power, I ∝ *P^n^*, where *P* denotes the near-infrared pump intensity and the exponent *n* corresponds to the number of excitation quanta required to populate the emitting level [18,19,63]. Values of *n* between 1 and 2 usually indicate two-photon processes affected by saturation or cross-relaxation, whereas values approaching 3 indicate three-owhoton processes. The observed emissions are generally attributed to cooperative sensitization or to stepwise energy migration from excited Yb^3+^ pairs to Ln^3+^ acceptor ions. The reduction in CL in codoped systems suggests that energy transfer to Ln^3+^ ions competes efficiently with radiative CL from Yb^3+^ pairs [18,19,20,21,22,23,24,25,31,32,33,34].

### 3.2. Downconverted Yb^3+^ Emission Band

**Figure 8:**
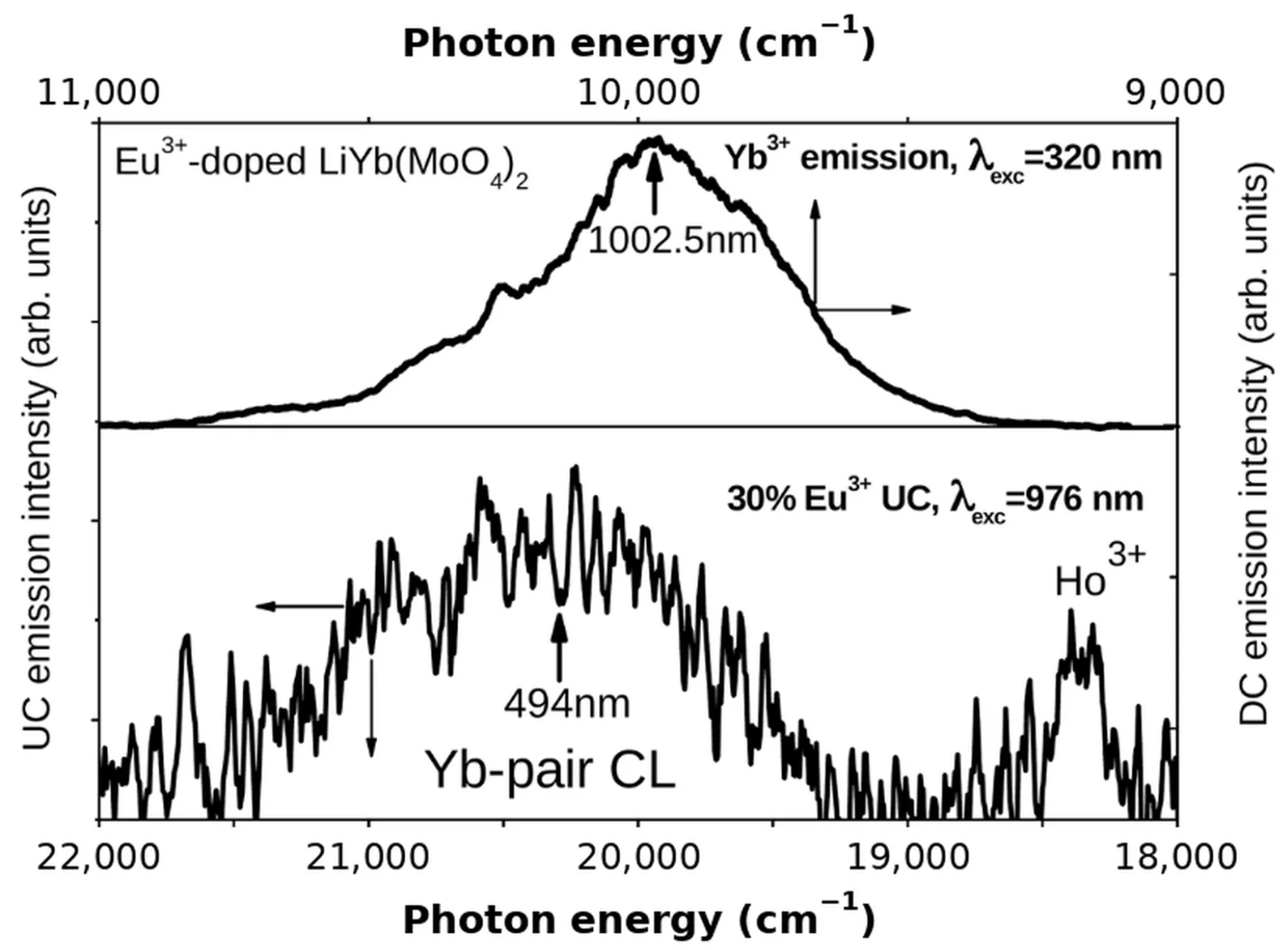
Cooperative-luminescence spectrum from LiYb_0.695_Eu_0.30_Ho_0.005_(MoO_4_)_2_ compared on an energy scale with its near-infrared Yb^3+ 2^F_5/2_ → ^2^F_7/2_ emission. A weak Ho^3+^ band is also present.

Yb^3+^ has the outer-electron configuration 4f^13^5s^2^5p^6^, with a ^2^F_7/2_ ground state and only one excited multiplet, ^2^F_5/2_ [14,15,16,17]. The shielding provided by the filled 5s^2^ and 5p^6^ subshells renders the 4f electrons largely insensitive to the host matrix, except for the Stark splitting that emerges in non-centrosymmetric crystal fields. At low temperature, the ^2^F_5/2_ → ^2^F_7/2_ emission and ^2^F_7/2_ → ^2^F_5/2_ absorption spectra can show several sharp Stark components [14,82,89]. At room temperature, however, vibronic coupling and thermal broadening often merge these components into a broad near-infrared emission band around 1000 nm, as illustrated for LiYb(MoO_4_)_2_-based crystals in Figure 8 [82,89,90].

### 3.3. Stokes and Anti-Stokes Luminescence Bands in Er^3+^/Yb^3+^-Doped Oxyfluoride Glass-Ceramics

**Figure 9:**
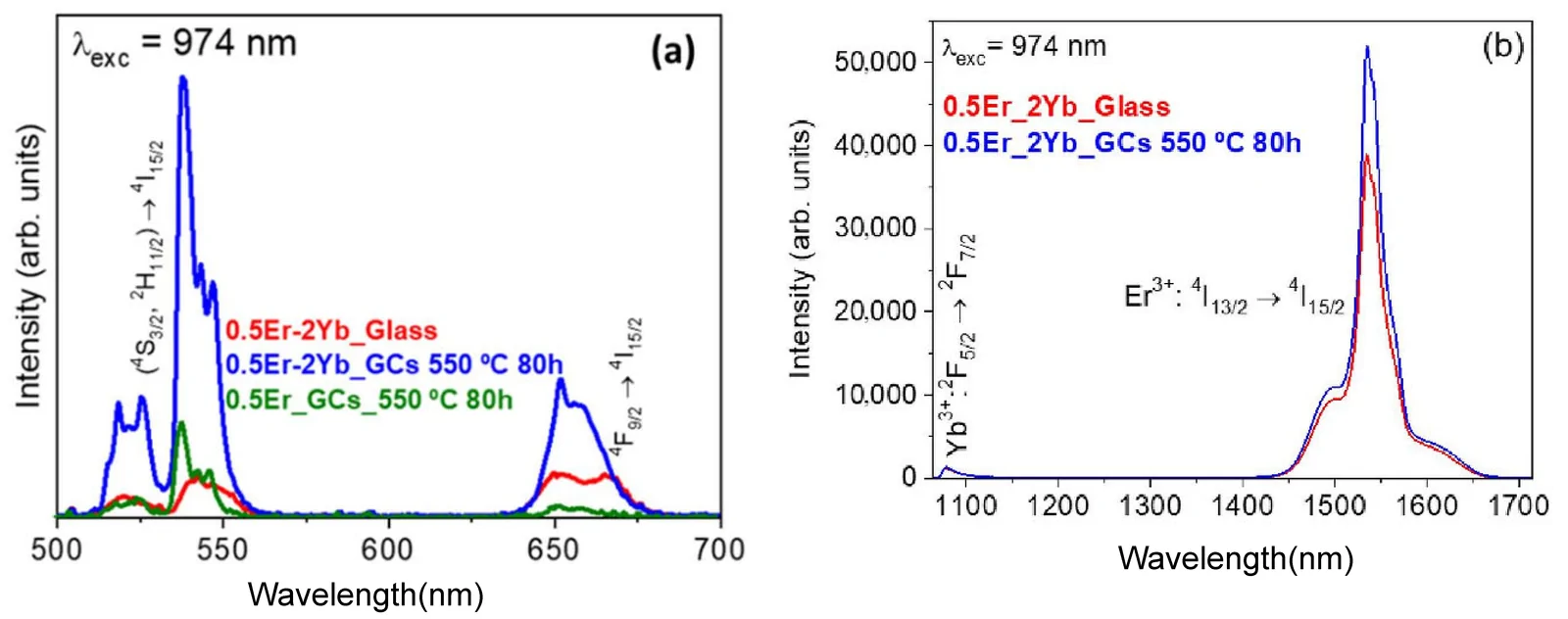
Representative upconversion and near-infrared emissions of Er^3+^/Yb^3+^-doped NaGdF_4_-containing oxyfluoride glass-ceramics. (**a**) Visible upconversion emission spectra of Er^3+^-doped and Er^3+^/Yb^3+^-codoped 70Si7Gd precursor glasses and glass-ceramics heat-treated at 550 °C for 80 h under 974 nm excitation. (**b**) Near-infrared emission spectra of the Er^3+^/Yb^3+^-codoped precursor glass and corresponding glass-ceramic under 974 nm excitation, showing the Yb^3+ 2^F_5/2_ → ^2^F_7/2_ emission near 1.0 μm and the Er^3+^ ^4^I_13/2_ → ^4^I_15/2_ emission around 1.5 μm. Adapted from Ref. [91] under the terms of the Creative Commons Attribution 4.0 International License (CC BY 4.0). The original panels were reformatted and rearranged for presentation without alteration of the underlying spectral data.

Representative Stokes and anti-Stokes emissions from Er^3+^/Yb^3+^-doped oxyfluoride glass-ceramics are shown in Figure 9. The spectra were obtained from NaGdF_4_-containing precursor glasses and glass-ceramics under 974 nm excitation [91]. In the visible region, the characteristic Er^3+^ upconversion emissions are assigned to the ^2^H_11/2_, ^4^S_3/2_ → ^4^I_15/2_ green transitions and the ^4^F_9/2_ → ^4^I_15/2_ red transition. In the near-infrared region, the emission features are associated mainly with the Yb^3+ 2^F_5/2_ → ^2^F_7/2_ transition near 1.0 μm and the Er^3+^ ^4^I_13/2_ → ^4^I_15/2_ transition around 1.5 μm. Compared with the precursor glass, the heat-treated glass-ceramic exhibits enhanced and better-resolved emission features, which supports the incorporation of Er^3+^ and Yb^3+^ ions into the low-phonon-energy NaGdF_4_ nanocrystals.

Analogous simultaneous Stokes and anti-Stokes luminescence was reported for Er^3+^/Yb^3+^-codoped Pb_4_Yb_3_F_17_-containing oxyfluoride nanoglass-ceramics under 940 nm excitation [66]. Although the corresponding experimental spectrum is not reproduced here, the observation of Er^3+^ emissions under the preferential excitation of Yb^3+^ provides evidence for efficient Yb^3+^ → Er^3+^ energy transfer in this system.

**Figure 10:**
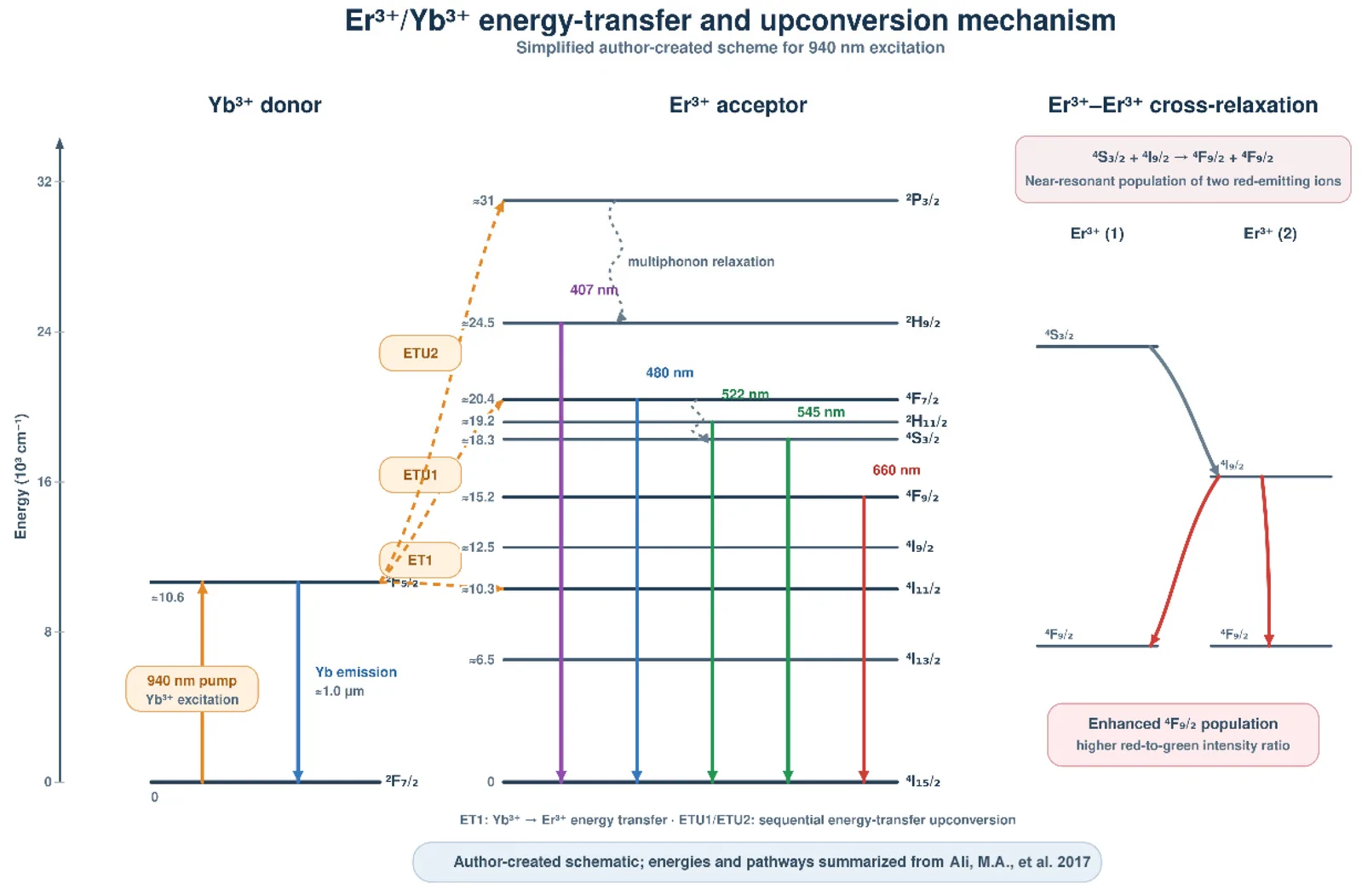
Author-created simplified energy-level scheme illustrating Yb^3+^-sensitized Er^3+^ upconversion under excitation within the Yb^3+^ absorption band. The diagram shows Yb^3+^ excitation, Yb^3+^ → Er^3+^ energy transfer to the ^4^I_11/2_ level, sequential energy-transfer upconversion to the ^4^F_7/2_ and higher-lying Er^3+^ levels, multiphonon relaxation, and the principal visible Er^3+^ emissions. The near-resonant Er^3+^–Er^3+^ cross-relaxation process ^4^S_3/2_ + ^4^I_9/2_ → ^4^F_9/2_ + ^4^F_9/2_ increases the population of the red-emitting ^4^F_9/2_ level [41].

The observation of Er^3+^ emission under excitation near the Yb^3+^ absorption band indicates efficient Yb^3+^ → Er^3+^ energy transfer. The initial resonant energy-transfer step can be expressed as ^4^I_15/2_ (Er^3+^)+ ^2^F_5/2_ (Yb^3+^)→^4^I_11/2_ (Er^3+^)+ ^2^F_7/2_ (Yb^3+^). A subsequent energy-transfer-upconversion step, ^4^I_11/2_ (Er^3+^)+ ^2^F_5/2_ (Yb^3+^)→^4^F_7/2_ (Er^3+^)+ ^2^F_7/2_ (Yb^3+^), populates the higher-lying Er^3+^ manifold. Multiphonon relaxation from ^4^F_7/2_ then populates the ^2^H_11/2_, ^4^S_3/2_, and ^4^F_9/2_ levels, giving rise to the characteristic green and red upconversion emissions. These principal excitation, energy-transfer, relaxation, and emission pathways are summarized in the author-created energy-level scheme shown in Figure 10.

Figure 10 presents an author-created simplified energy-level scheme for Yb^3+^-sensitized Er^3+^ upconversion in oxyfluoride glasses and glass-ceramics. The relevant Er^3+^ levels are located at approximately 10,000–10,500 cm^−1^ for 4I_11/2_, 12,500 cm^−1^ for ^4^I_9/2_, 15,200 cm^−1^ for ^4^F_9/2_, 18,300 cm^−1^ for ^4^S_3/2_, and 20,400 cm^−1^ for ^4^F_7/2_. In addition to sequential Yb^3+^ → Er^3+^ energy transfer, Er^3+^–Er^3+^ cross-relaxation can influence the relative intensities of the green and red emissions. In particular, the near-resonant process ^4^S_3/2_(Er^3+^) + ^4^I_9/2_(Er^3+^) → ^4^F_9/2_(Er^3+^) + ^4^F_9/2_(Er^3+^) increases the population of the red-emitting ^4^F_9/2_ level. This process can therefore contribute to an increase in the red-to-green intensity ratio, R_r/g_.

In the Pb_4_Yb_3_F_17_-containing oxyfluoride nanoglass-ceramic investigated in Ref. [66], a weak upconversion emission was additionally observed at approximately 480 nm and was assigned to the Er^3+^ ^4^F_7/2_ → ^4^I_15/2_ transition. The ^4^F_7/2_ level can be populated through a two-step Yb^3+^-sensitized process: an initial Yb^3+^ → Er^3+^ energy-transfer step populates the Er^3+^ ^4^I_11/2_ level, followed by a second energy-transfer-upconversion step to ^4^F_7/2_. The emission near 407 nm was assigned to the ^2^H_9/2_ → ^4^I_15/2_ transition. Population of ^2^H_9/2_ may involve an additional Yb^3+^-sensitized upconversion step to a higher-lying Er^3+^ level, such as ^2^P_3/2_, followed by multiphonon relaxation to ^2^H_9/2_. These proposed pathways are included in the generalized mechanism shown in Figure 10.

## 4. Design and Characterization of Yb^3+^-Containing Glass-Ceramics

### 4.1. Nucleation, Phase Fraction, and Optical Transparency

The objective is to achieve a controlled population of crystals dispersed in a continuous glassy matrix—not merely to detect an XRD signal. Scattering behavior is governed by four interrelated factors: crystalline fraction, grain-size distribution, spatial uniformity, and refractive-index mismatch. When crystallites are significantly smaller than the wavelength of light, high transparency can be retained; however, aggregation or a wide size distribution may still introduce optical losses. Consequently, heat-treatment protocols must be optimized with respect to at least two performance metrics—crystallization and transparency—rather than against a single parameter such as peak intensity [92,93,94,95,96,97,98,99].

Rare-earth partitioning introduces a third consideration. Luminescence may be enhanced when Yb^3+^ and an activator ion are incorporated into a low-phonon fluoride nanocrystal, yet the high local concentration within such a confined phase can also promote cross-relaxation or clustering. Meanwhile, the composition of the residual glass evolves as crystallization proceeds, altering its viscosity, refractive index, and rare-earth solubility over the course of the same thermal schedule. These coupled effects explain why the most prolonged or highest-temperature anneal is rarely the optimum choice.

The crystalline fraction should ideally be quantified via Rietveld refinement using an internal standard or through a calibrated complementary technique. The Scherrer estimate derived from XRD peak broadening represents the coherent domain size, which does not necessarily correspond to the actual particle diameter observed in SEM. Reporting both quantities—along with the instrument-broadening correction and a size distribution extracted from imaging—helps prevent apparent discrepancies from being misinterpreted as evidence of a novel physical effect.

### 4.2. Capabilities and Limitations of Characterization Methods

DSC/DTA identifies thermal events and supports kinetic analysis, but overlapping exotherms and baseline choices can obscure multiple phases. XRD is phase-specific for sufficiently ordered and abundant crystals, yet very small, strained, or low-fraction nanocrystals may produce peaks too broad or weak for confident detection. TEM with selected-area electron diffraction can reveal such nanocrystals and distinguish surface from volume populations, although its field of view may not represent the bulk. Raman spectroscopy is sensitive to short-range network reorganization and can detect vibrational signatures unavailable from XRD, but band overlap and fluorescence backgrounds complicate quantification. A combined XRD–TEM–Raman strategy has directly demonstrated heat-treatment-induced NaYF_4_ nanocrystals and associated network changes in Ho^3+^/Yb^3+^ oxyfluoride glass-ceramics [100].

XANES can test oxidation state and near-edge coordination changes, whereas EXAFS yields average neighbor identities, distances, and coordination numbers around Yb. These methods are particularly valuable when Yb-rich clusters or nanophases are below the XRD detection limit; EXAFS has resolved the local environment and phosphate association of Yb^3+^ in phase-separated silica-based preforms [101]. Their limitation is ensemble averaging, so coexisting Yb sites may require model comparison or complementary spectroscopy. Photoluminescence, lifetime, and quantum-yield measurements probe the functional consequence of site changes, but optical enhancement alone is not proof of crystal incorporation. The strongest mechanistic claim is obtained when thermal, diffraction, imaging, local-structure, and optical evidence converge on the same sequence.

## 5. Representative Ln^3+^-Yb^3+^ Codoped Glass-Ceramics

### 5.1. Er^3+^- Yb^3+^ Codoped Fluoro-Aluminosilicate Glass-Ceramics

In 45SiO_2_-15Al_2_O_3_-12Na_2_O-21BaF_2_-7YF_3_-0.5ErF_3_-1YbF_3_ glass codoped with Er^3+^ and Yb^3+^, controlled heat treatment produces cubic BaYF_5_ nanocrystals [62]. That study is instructive because it connects phase formation, microstructure, transmission, and upconversion. Yb^3+^ absorbs near 980 nm and sensitizes Er^3+^, while the fluoride nanophase provides a lower-phonon environment than the oxide-rich matrix.

**Figure 11:**
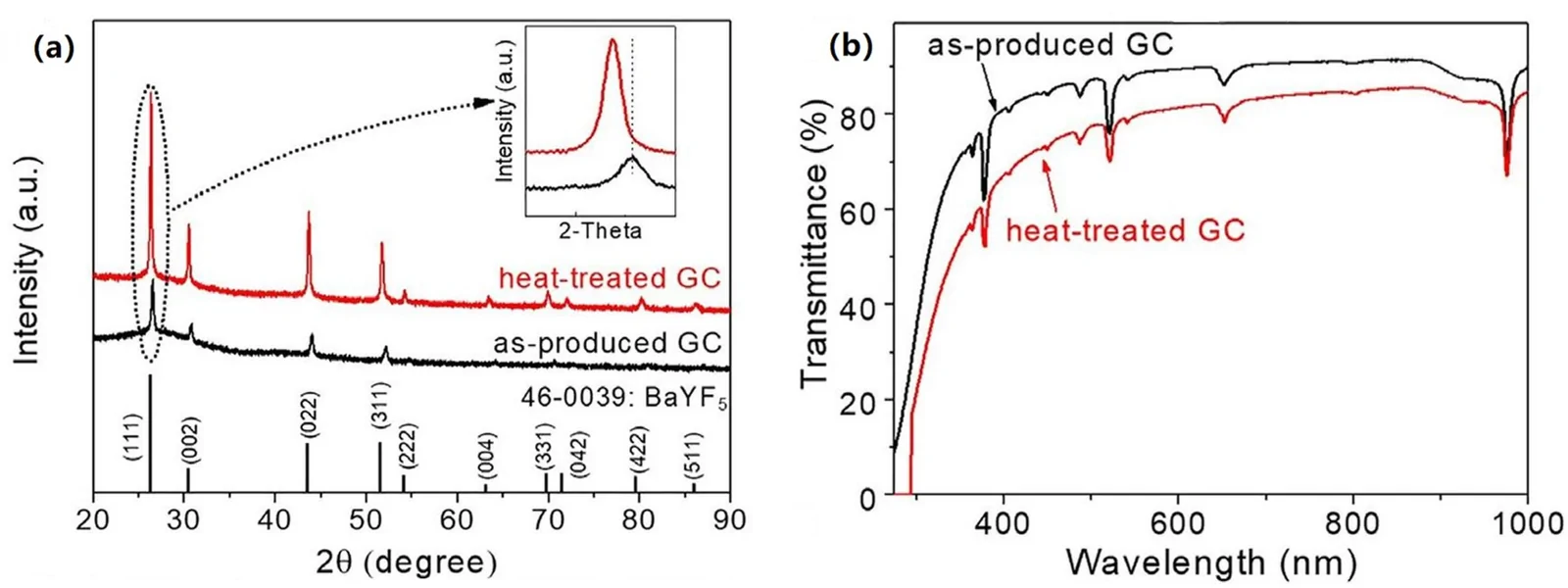
(**a**) XRD patterns of as-produced and heat-treated glass-ceramics with the cubic BaYF_5_ reference; (**b**) corresponding transmission spectra. Reproduced with permission from Ref. [62]. Copyright 2021, John Wiley and Sons.

**Figure 12:**
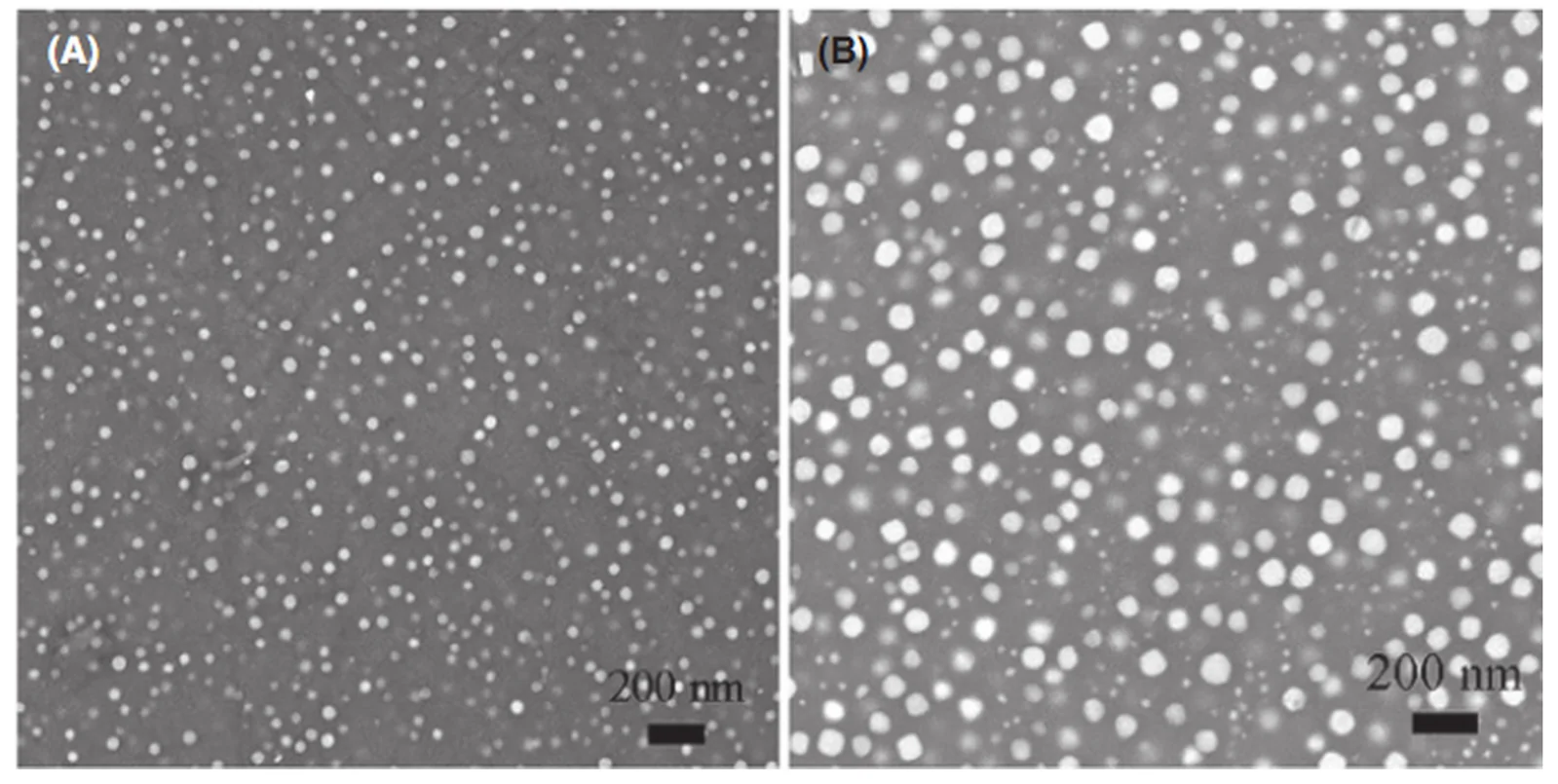
SEM micrographs of the as-produced (**A**) and heat-treated (**B**) Er^3+^–Yb^3+^ fluoro-aluminosilicate glass-ceramics. Reproduced with permission from Ref. [62]. Copyright 2021, John Wiley and Sons.

XRD establishes the crystalline phase, and transmission above 80% indicates that the heat-treated microstructure remains optically homogeneous on the wavelength scale (Figure 11). The transmittance spectra of both as-produced and heat-treated GC samples (measured over 275–1000 nm) provide complementary information to the Raman data. In Figure 11b, absorption features are observed at approximately 378, 487, 523, 541, 653, and 976 nm. The first five of these correspond to Er^3+^ transitions from the ^4^I_15/2_ ground state to the ^4^G_11/2_, ^4^F_7/2_, ^2^H_11/2_, ^4^S_3/2_, ^4^F_9/2_ excited levels, while the 976 nm line arises from the Yb^3+ 2^F_7/2_ → ^2^F_5/2_ transition. Notably, no absorption feature attributable to the BaYF_5_ crystalline phase is detected. This absence is consistent with the small dimensions of the precipitated BaYF_5_ nanocrystals—approximately 10 nm as shown in Figure 12B—which are substantially smaller than the wavelengths of visible light. This size regime explains why both the as-produced and heat-treated GC samples maintain high transmittance (>80%). By contrast, the presence of larger BaYF_5_ crystallites would be expected to introduce significant scattering losses and thereby reduce transparency.

Electron microscopy showed approximately 25 nm domains in the as-produced material and particles near 60–65 nm after heat treatment, together with smaller newly precipitated domains (Figure 12) [62]. The XRD coherent-domain estimate and the image-based particle size are similar in scale but should not be treated as identical quantities. The coexistence of sizes suggests that nucleation continued while earlier particles grew. 

This observation is mechanistically more informative than a single average size. Continued nucleation broadens the population and can preserve transparency if both populations remain nanoscale; excessive growth would increase scattering. Rare-earth partitioning into BaYF_5_ was inferred from the enhanced emission and crystal-field features, but a quantitative Yb^3+^ or Er^3+^ partition coefficient was not reported.

The case therefore supports a coupled design rule: annealing should maximize the fraction of rare-earth ions in the desired nanophase before particle coarsening compromises transmission. Quantitative phase fraction, lifetime, and absolute quantum efficiency would be needed to compare this optimum with other glass families.

#### UC Luminescent Behavior

One advantage of controlled glass crystallization is that it can place rare-earth ions into low-phonon-energy crystalline environments while maintaining the mechanical stability and processability of the glass matrix [92,93,94,95,96,97,98,99]. In Er^3+^–Yb^3+^ codoped systems, Yb^3+^ acts as a sensitizer under 940–980 nm excitation and transfers energy to Er^3+^, leading to population of the ^4^I_11_/_2_, ^4^F_7/2_, ^2^H_11/2_, ^4^S_3/2_, and ^4^F_9/2_ levels. The resulting green and red emissions are strongly affected by crystallite size, rare-earth partitioning, phonon energy, and cross-relaxation pathways [18,19,20,34,62,92,93,94,95,96,97].

**Figure 13:**
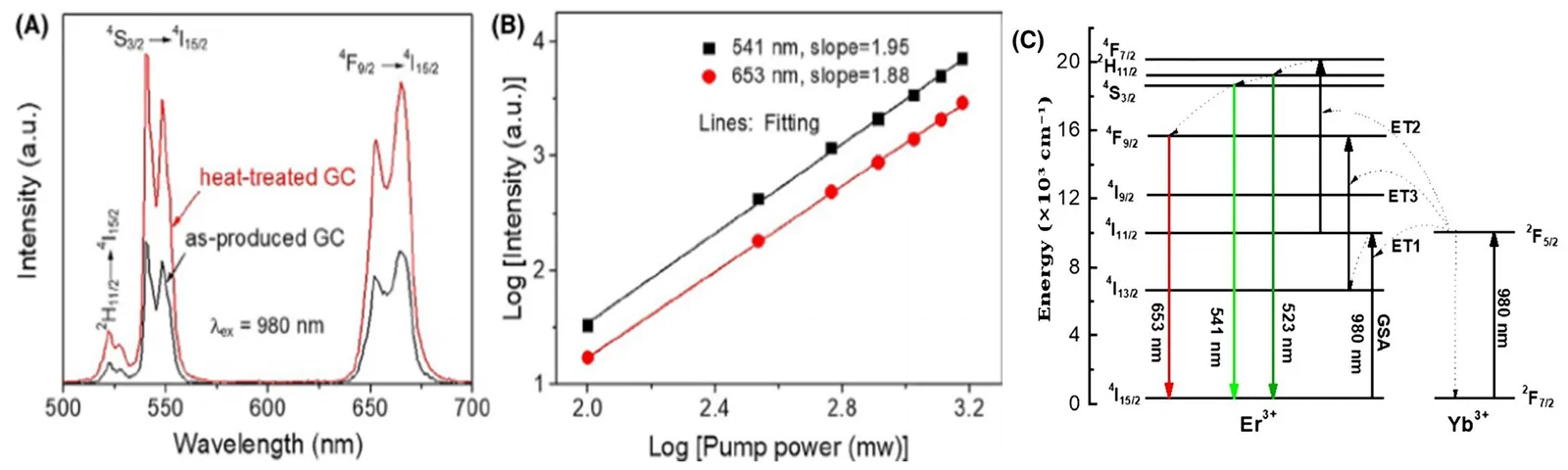
(**A**) Upconversion spectra of as-produced and heat-treated glass-ceramics; (**B**) pump-power dependence for the heat-treated sample; (**C**) proposed Er^3+^–Yb^3+^ energy-transfer pathways under 980 nm excitation. Reproduced with permission from Ref. [62]. Copyright 2021, John Wiley and Sons.

Figure 13A presents the upconversion (UC) emission spectra of Er^3+^–Yb^3+^ codoped GC samples—both as-produced and heat-treated—under 980 nm excitation, recorded over the 500–700 nm range. Three characteristic emission peaks appear at 523 nm (^2^H_11/2_ → ^4^I_15/2_), 541 nm (^4^S_3/2_ → ^4^I_15/2_), and 653 nm (^4^F_9/2_ → ^4^I_15/2_). A notable feature is the distinct Stark splitting observed in both samples, particularly the resolved doublet at ~541 nm and ~549 nm within the ^4^S_3/2_ → ^4^I_15/2_ band. This splitting originates from the crystal-field-induced lifting of degeneracy in the ^4^I_15_/_2_ ground state, given that the emitting level is the lowest relaxed component of the ^4^S_3/2_ manifold even at room temperature.

The heat-treated GC exhibits approximately twice the UC intensity of its as-produced counterpart. This enhancement is attributed to the preferential incorporation of Er^3+^ ions into the BaYF_5_ fluoride nanocrystals, where the lower phonon energy environment suppresses non-radiative multiphonon relaxation. The UC emission intensity (IUC) generally scales with excitation power (*P*) according to IUC ∝ *P^n^*, where *n* denotes the number of 980 nm photons required to populate the emitting states (^2^H_11/2_, ^4^S_3/2_, or ^4^F_9/2_). Because *n* ≥ 2, the process corresponds to anti-Stokes emission. Figure 13B shows the logarithmic plot of IUC versus *P*; the fitted slopes are 1.95 for the 541 nm band and 1.88 for the 653 nm band, confirming that a two-photon mechanism dominates the UC process.

The underlying energy-transfer scheme is illustrated in Figure 13C. Upon 980 nm excitation, Yb^3+^ ions are initially promoted from the ^2^F_7/2_ ground state to the ^2^F_5/2_ excited level via ground-state absorption (GSA). These excited Yb^3+^ ions then relax non-radiatively, transferring energy to adjacent Er^3+^ ions and promoting them from ^4^I_15/2_ to ^4^I_11/2_ through a resonant ET1 process: ^4^I_15/2_ (Er^3+^) + ^2^F_5/2_ (Yb^3+^) → ^4^I_11/2_ (Er^3+^) + ^2^F_7/2_ (Yb^3+^). In parallel, Er^3+^ ions can also undergo direct GSA from ^4^I_15_/_2_ to ^4^I_11_/_2_. A subsequent ET2 step—^4^I_11/2_ (Er^3+^) + ^2^F_5/2_ (Yb^3+^) → ^4^F_7/2_ (Er^3+^) + ^2^F_7/2_ (Yb^3+^)—drives the system to the ^4^F_7/2_ level. From there, non-radiative relaxation populates the ^2^H_11/2_ and ^4^S_3/2_ emissive states, which then decay radiatively to ^4^I_15/2_, producing the 523 and 541 nm emissions. The 653 nm red emission (^4^F_9/2_ → ^4^I_15/2_) can be populated via two pathways: (i) non-radiative relaxation from ^4^S_3/2_, or (ii) an ET3 process—^4^I_13/2_ (Er^3+^) + ^2^F_5/2_ (Yb^3+^) → ^4^F_9/2_ (Er^3+^) + ^2^F_7/2_ (Yb^3+^)—following non-radiative decay from ^4^I_11/2_.

To summarize the UC behavior: the heat-treated GC shows a twofold enhancement over the as-produced GC, attributable to the more effective partitioning of both Er^3+^ and Yb^3+^ into the low-phonon BaYF_5_ environment. Notably, the heat-treated material remains stable against further crystallization, ensuring stable UC performance at operating temperatures, and exhibits promising temperature-sensing characteristics.

Attribution of the upconverted 523, 541, and 653 nm emissions to the two-photon excitation process under 980 nm LD excitation is also explained as follows. The 980 nm LD irradiation to the heat-treated GC sample of 45SiO_2_-15Al_2_O_3_ -12Na_2_O-21BaF_2_-7YF_3_-0.5ErF_3_-1YbF_3_ gives rise to the excitation of not only the Yb^3+^ of YbF_3_ but also the Er^3+^ of ErF_3_ because the two ions have a gap energy of ~10,206 cm^−1^ (corresponding to 980 nm). For instance, the energy separation between the ^4^I_11/2_ and ^4^I_15/2_ levels of Er^3+^ is approximately 10,000 cm^−1^, which matches the energy of a 980 nm photon and thus allows excitation from the ground state to the ^4^I_11/2_ manifold. From this intermediate level, a second 980 nm photon can promote the ion further to the ^4^F_7/2_ state, as the ^4^F_7/2_-^4^I_11/2_ gap (~11,000 cm^−1^) is also resonant with the excitation wavelength. Consequently, Er^3+^ can be raised to the ^4^F_7/2_ level through a two-photon absorption sequence. Subsequent non-radiative multiphonon relaxation then populates the lower-lying ^2^H_11/2_, ^4^S_3/2_, and ^4^F_9/2_ emitting states, which give rise to the 523, 541, and 653 nm emissions, respectively (Figure 13C).

An alternative two-photon pathway involves the initial excitation of Yb^3+^ from ^2^F_7/2_ to ^2^F_5/2_ by a 980 nm photon. The excited Yb^3+^ ion subsequently undergoes spontaneous ^2^F_5/2_→^2^F_7/2_ emission at ~980 nm, and this emitted photon can then be reabsorbed by an Er^3+^ ion to promote it from ^4^I_11/2_ to ^4^F_7/2_. This means that two 980 nm photons are used to excite into the ^4^F_7/2_ state, followed by the generation of the 523, 541, and 653 nm emissions finally.

### 5.2. Tb^3+^–Ho^3+^–Yb^3+^-Codoped Glass-Ceramics

**Figure 14:**
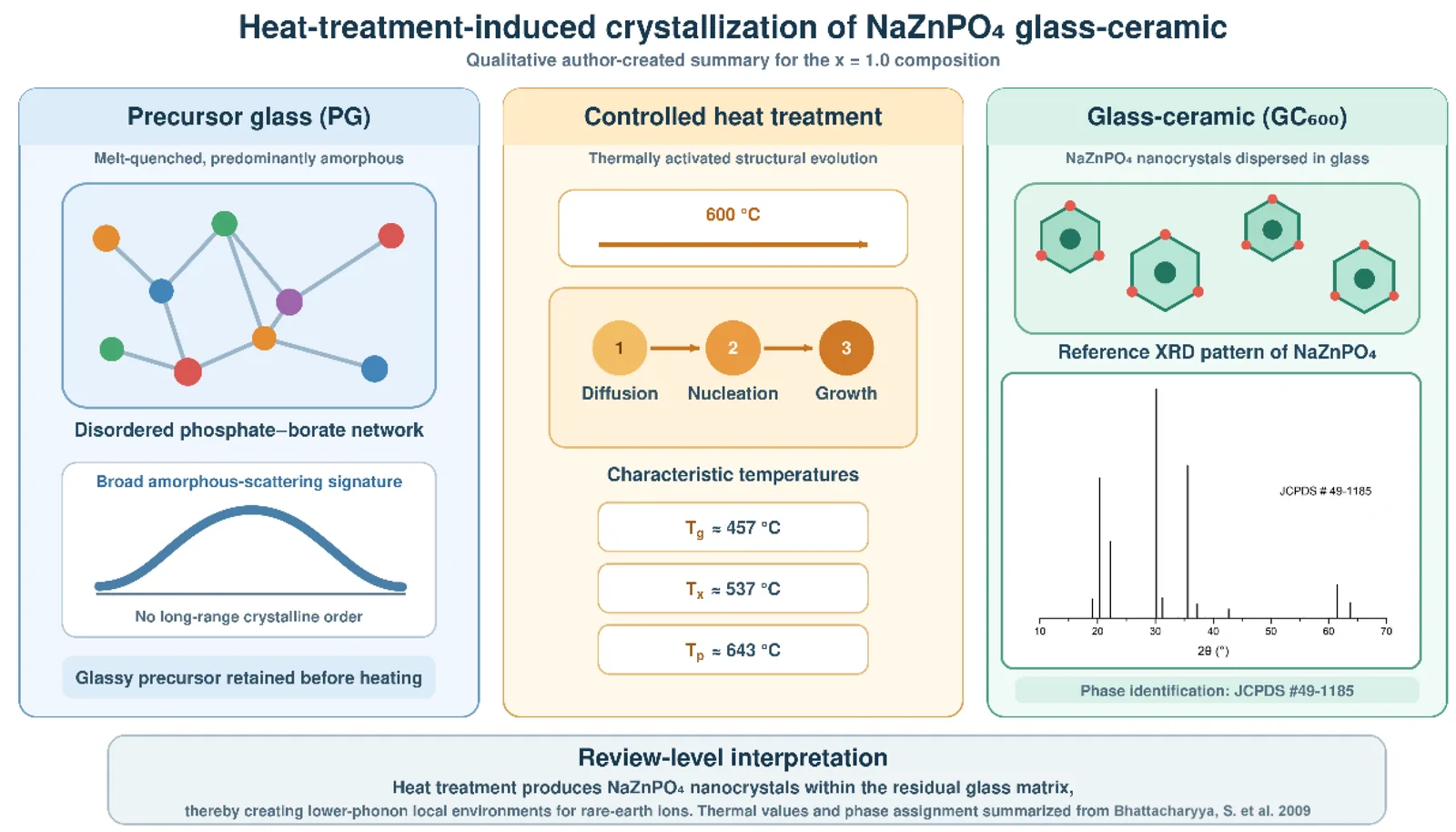
Author-created schematic summarizing the heat-treatment-induced conversion of the 20Na_2_O-42ZnO-28P_2_O_5_-10B_2_O_3_-2.0Yb_2_O_3_-0.15Ho_2_O_3_-1.0Tb_4_O_7_ precursor glass (PG) into the NaZnPO_4_-containing glass-ceramic (GC_600_). The thermal parameters and phase assignment are summarized from Refs. [38,63].

Phosphate glasses with compositions (in mol %) consisting of seven constituents of 20Na_2_O-42ZnO-28P_2_O_5_-10B_2_O_3_-2.0Yb_2_O_3_-0.15Ho_2_O_3_-*x*Tb_4_O_7_ (with *x* = 0.6, 0.8, 1.0, 1.2) were melted, annealed, and subsequently heat-treated at 600 °C to form a transparent NaZnPO_4_-containing glass-ceramic (GC_600_) [63]. The reported thermal markers were T_g_ ≈ 457 °C, onset T_x_ ≈ 537 °C, and a crystallization peak near 643 °C. These values define a processing window, but they do not alone identify whether nucleation began at T_g_ or quantify the crystalline fraction. The reported XRD results distinguish the predominantly amorphous precursor glass (PG) from the NaZnPO_4_-containing GC_600_ product [63]; the corresponding heat-treatment-induced phase evolution is summarized schematically in Figure 14. Yb^3+^ supplies near-infrared sensitization, while Ho^3+^ and Tb^3+^ provide multiple visible emitting levels. The system therefore tests how crystallization changes a multi-ion transfer network, but concentration quenching and cross-relaxation must be separated from the effect of the crystalline host.

The crystallization behavior and upconversion optical properties of NaZnPO_4_:Yb^3+^/Tb^3+^/Ho^3+^ nanocrystals embedded in a glass matrix have been investigated in multicomponent systems [63]. These studies are important because Tb^3+^ and Ho^3+^ introduce additional green, red, and near-infrared emitting levels, while Yb^3+^ provides efficient sensitization under near-infrared excitation. Such multi-ion systems allow emission color tuning through energy transfer, cooperative sensitization, and cross-relaxation processes [18,19,20,31,32,33,34,63,67].

#### 5.2.1. Crystallization and Upconversion Spectra

**Figure 15:**
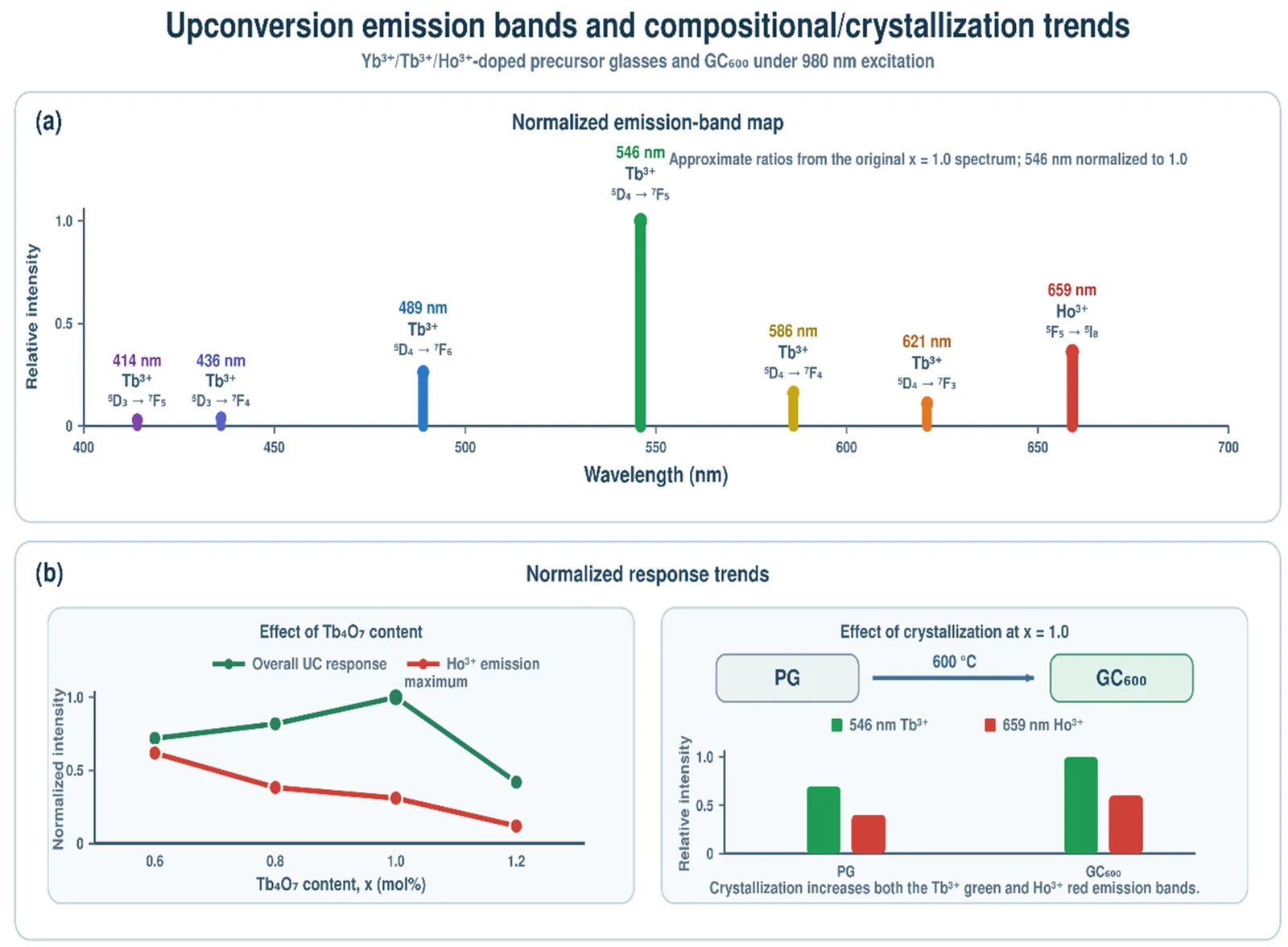
Author-created summary of the upconversion behavior of Yb^3+^/Tb^3+^/Ho^3+^-doped precursor glasses and the corresponding GC_600_ glass-ceramic under 980 nm excitation: (**a**) principal Tb^3+^ and Ho^3+^ emission bands and their transition assignments; (**b**) qualitative effects of Tb_4_O_7_ content and crystallization, showing the maximum overall UC response at x = 1.0 mol%, concentration quenching at x = 1.2 mol%, weakening of the Ho^3+^ 659 nm emission with increasing x, and enhanced emission following the conversion of PG into GC_600_. The wavelengths, assignments, and qualitative trends are summarized from Ref. [63].

The PG samples with nominal composition 20Na_2_O-42ZnO-28P_2_O_5_-10B_2_O_3_-2.0Yb_2_O_3_-0.15Ho_2_O_3_-*x*Tb_4_O_7_ with *x* = 0.6, 0.8, 1.0 and 1.2 mol% were excited by a 980 nm laser. The principal upconversion emission bands and their transition assignments are summarized in Figure 15a. Five intense bands are observed at 489, 546, 586, 621, and 659 nm. The first four are assigned to Tb^3+^ transitions (^5^D_4_→^7^F_6_, ^7^F_5_, ^7^F_4_, and ^7^F_3_), while the 659 nm band corresponds to the ^5^F_5_→^5^I_8_ transition of Ho^3+^. Two weaker violet emissions at 414 and 436 nm are also attributable to Tb^3+^ [63].

Among the four nominal Tb_4_O_7_ contents investigated, the strongest UC emission is observed for the sample with x = 1.0 mol%. Beyond this level, concentration quenching sets in and the Tb^3+^-related emission intensity declines. Concurrently, the Ho^3+^ emission intensity progressively decreases as the Tb^3+^ content rises from 0.6 to 1.2 mol%, which suggests a Ho^3+^ → Tb^3+^ energy-transfer pathway [63]. These qualitative composition-dependent trends are summarized in Figure 15b.

In comparison with the PG precursor, the GC_600_ sample derived from the same composition (x = 1.0 mol%) exhibits a substantially enhanced UC emission, as summarized in Figure 15b. This enhancement was attributed to the crystallization of NaZnPO_4_ nanocrystals with low phonon energy, which effectively suppresses non-radiative multiphonon relaxation and thereby promotes radiative transitions [63].

#### 5.2.2. Two-Photon Excitation Process for the UC Emission

**Figure 16:**
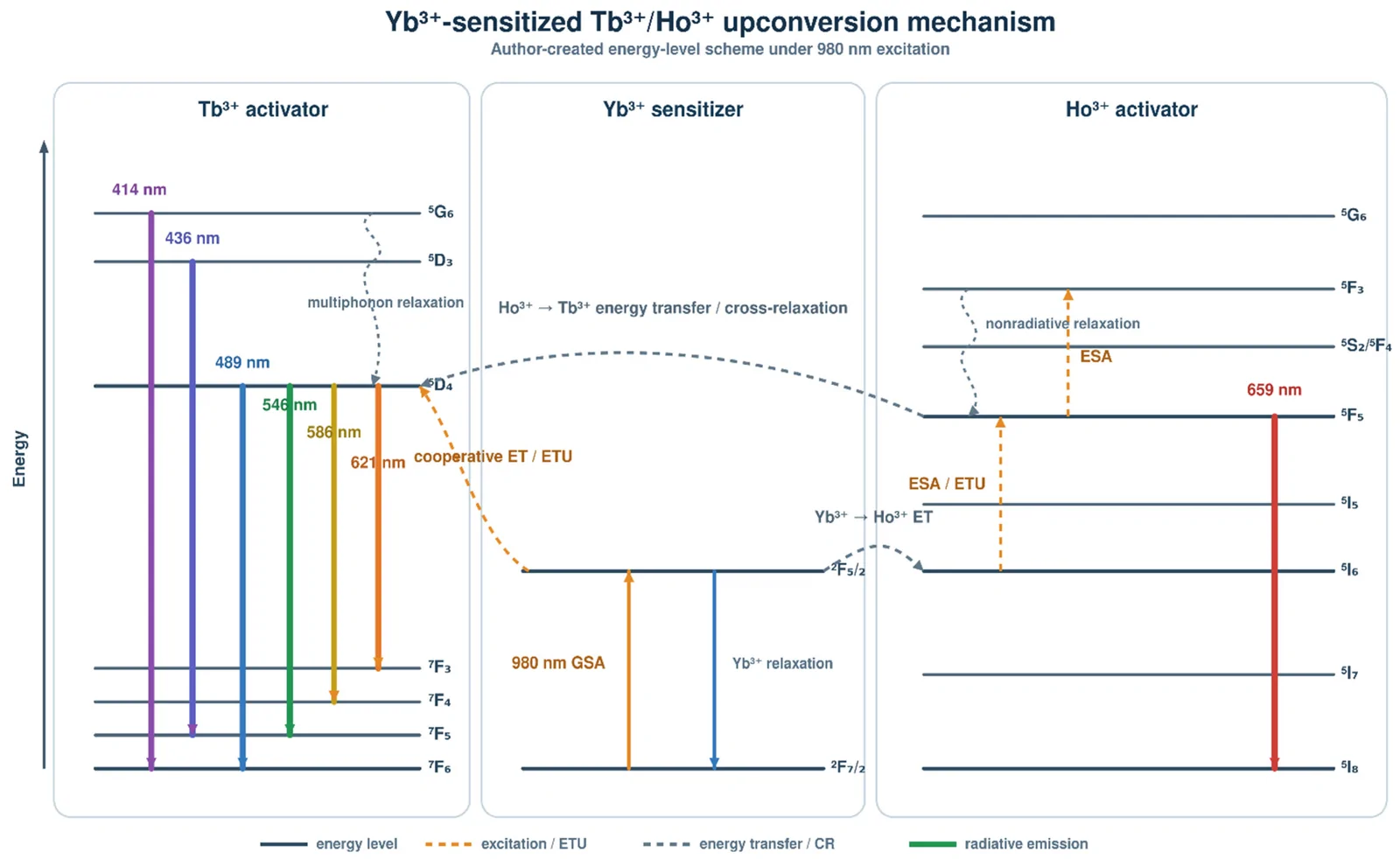
Author-created energy-level scheme illustrating Yb^3+^-sensitized Tb^3+^/Ho^3+^ upconversion under 980 nm excitation, including ground-state absorption (GSA), energy transfer (ET), energy-transfer upconversion (ETU), excited-state absorption (ESA), cross-relaxation (CR), non-radiative relaxation, and the principal Tb^3+^ and Ho^3+^ emissions. The proposed pathways and emission assignments are summarized from Ref. [63].

The log–log-plotted UC emission intensities (IUC) vs. pump power (*P*) in the GC_600_ sample suggest that the slopes are 1.57 for the 546 nm green band (Tb^3+^: ^5^D_4_ → ^7^F_5_) and 1.42 for the 659 nm red band (Ho^3+^: ^5^F_5_ → ^5^I_8_). The log–log plot indicates that IUC is proportional to *P^n^* with *n* = 2, where *n* stands for the number of photons necessary to generate the UC emission, suggesting that a two-photon energy-transfer process with two 980 nm photons is involved to populate the ^5^D_4_ state of Tb^3+^ and also to populate the ^5^F_5_ state of Ho^3+^. The proposed Yb^3+^-sensitized pathways that populate the Tb^3+ 5^D_4_ and Ho^3+ 5^F_5_ emitting states are summarized in the author-created energy-level scheme shown in Figure 16.

## 6. Comparative Analysis of Crystallization Mechanisms Across Yb^3+^-Containing Glass Families

### 6.1. CaF_2_ Nanocrystallization in Oxyfluoride Glasses

A glass of composition 47.4SiO_2_-19Al_2_O_3_-28.4CaF_2_-2TbF_3_-3.2YbF_3_ (mol%) was fabricated by melting a homogeneous powder mixture of the constituent oxides and fluorides in a covered Pt-Rh crucible at 1400 °C for 1 h in air [64]. The resulting melt was cast onto a steel plate, covered with another plate, and left to cool gradually to ambient temperature.

**Figure 17:**
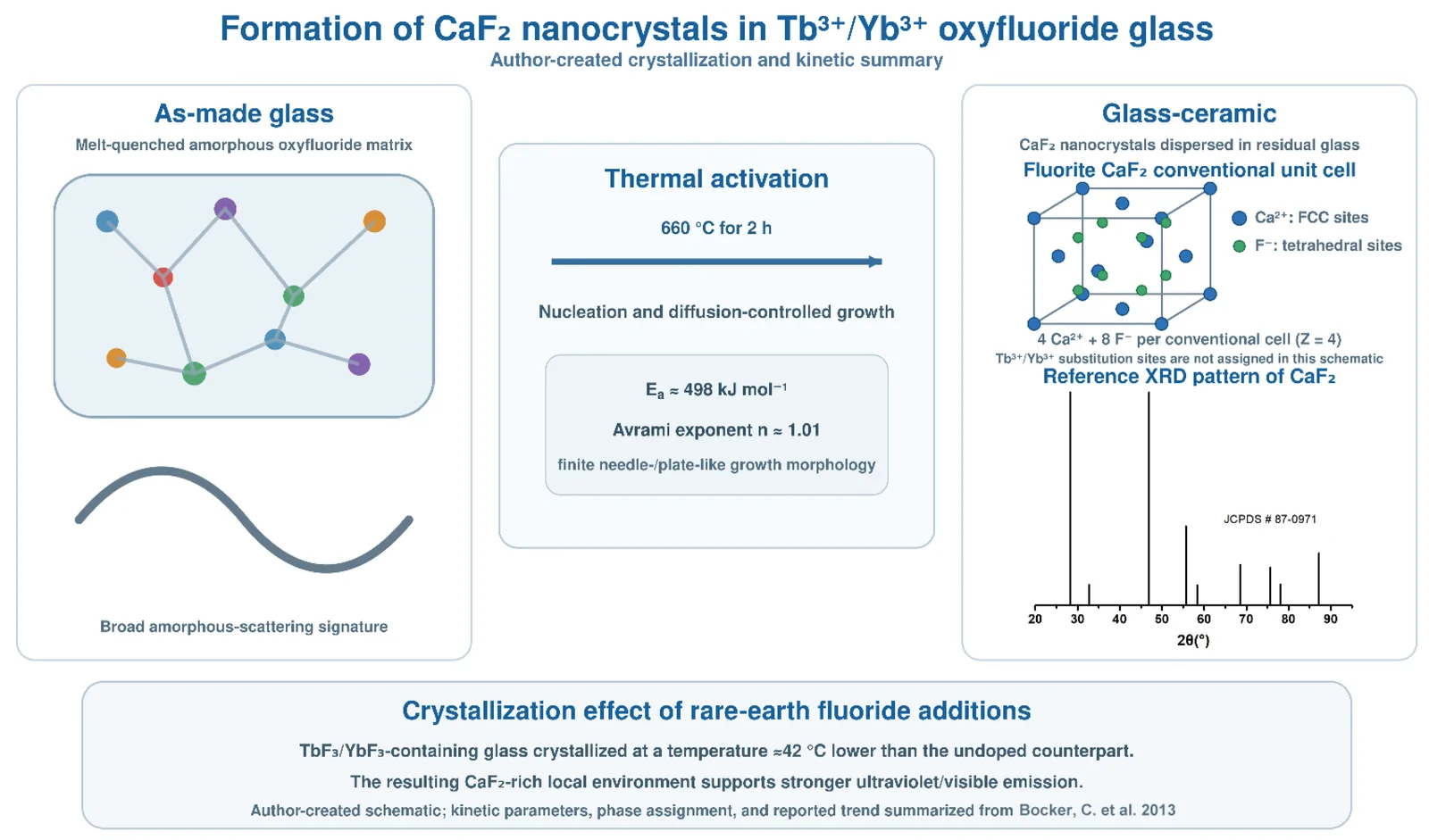
Author-created schematic summarizing the heat-treatment-induced formation of CaF_2_ nanocrystals in Tb^3+^/Yb^3+^-doped oxyfluoride glass at 660 °C for 2 h. The reported apparent activation energy, E_a_ ≈ 498 kJ mol^−1^, and Avrami exponent, *n* ≈ 1.01, indicate diffusion-controlled crystal growth. The kinetic parameters, phase assignment, and crystallization-temperature trend are summarized from Refs. [39,64].

The as-quenched product was fully amorphous, as expected for this glass-forming system. Subjecting it to a thermal treatment at 660 °C for 2 h triggered crystallization, producing a glass-ceramic in which CaF_2_ nanocrystals were detected by XRD [64]. The corresponding transformation from the amorphous precursor to the CaF_2_-containing glass-ceramic is summarized schematically in Figure 17. Undoped versions of the glass remained amorphous after quenching and could also be crystallized upon heating but only at temperatures some 42 °C higher than their Tb/Yb-containing counterpart. This difference demonstrates that the introduction of TbF_3_ and YbF_3_ effectively promotes crystallization at lower temperatures.

The crystallization kinetics were investigated by DSC, which yielded an apparent activation energy of ~498 kJ/mol and an Avrami exponent of *n* = 1.01. These values point toward a diffusion-governed growth regime, with crystallites adopting needle- and plate-like morphologies of limited length. Figure 17 summarizes both the reported kinetic descriptors and the heat-treatment-induced phase evolution, while transmission electron microscopy provided independent confirmation of the CaF_2_ nanocrystalline phase within the glass-ceramic matrix [64].

### 6.2. Cluster-Assisted Formation of Multicomponent Fluoride Phases

#### 6.2.1. Ca–Yb Fluoride Solid Solutions

The incorporation of Yb_2_O_3_ into NaF-CaF_2_-Al_2_O_3_-SiO_2_ glass does more than simply introduce an optically active ion. The trivalent cation can interact with fluoride-rich regions, maintain charge balance via local structural modifications, and be incorporated into a CaF_2_-derived lattice. This dual function—acting as a network modifier prior to crystallization and as a lattice constituent afterward—accounts for the observed dependence of the apparent activation energy and the resulting phase assemblage on Yb content [65].

Within the composition series examined, heat treatment yielded CaF_2_-related crystalline phases along with a Ca_0.8_Yb_0.2_F_2.2_ solid solution, with grain sizes in the range of 10–20 nm [65]. The apparent activation energy decreased monotonically with increasing Yb^3+^ loading. Because the crystalline product incorporates Yb, this trend is consistent with the idea that Yb-rich fluoride environments serve as precursors to the solid-solution phase.

This finding should not be overinterpreted as evidence that Yb^3+^ universally accelerates crystallization. Higher Yb content also modifies network connectivity, charge compensation mechanisms, and liquidus relationships. Disentangling catalytic nucleation from a shift in equilibrium phase stability requires compositionally matched series, quantitative phase-fraction analysis, and the local structural characterization of Yb prior to crystallization.

A related but lead-free example is provided by Yb^3+^/Er^3+^-codoped fluoro-borosilicate glasses, in which the partial replacement of SiO_2_ by B_2_O_3_ modifies the oxide–fluoride network and improves the selectivity of rare-earth-fluoride nanocrystallization [75]. In this system, Yb^3+^ acts not only as an optical sensitizer but also as a component of the precipitated KYb_2_F_7_ and KYb_3_F_10_ phases. The resulting phase evolution therefore provides direct evidence that rare-earth concentration, glass-network composition, and heat-treatment temperature jointly determine the identity of the nanocrystalline phase.

#### 6.2.2. Yb^3+^-Induced Rare-Earth-Fluoride Nanocrystallization in Fluoro-Borosilicate Glasses

An open-access example that demonstrates the coupled effects of glass composition, Yb^3+^ addition, and heat-treatment temperature was reported for nominal (60 − x)SiO_2_–xB_2_O_3_–20KF–20ZnF_2_ glasses (x = 0–40 mol%) [75]. YbF_3_ and ErF_3_ were introduced to provide 1.0 mol% Yb^3+^ and 0.2 mol% Er^3+^, respectively. Partial substitution of B_2_O_3_ for SiO_2_ reduced the glass-preparation temperature and altered the competition between the fluoride phases precipitated during subsequent heat treatment.

**Figure 18:**
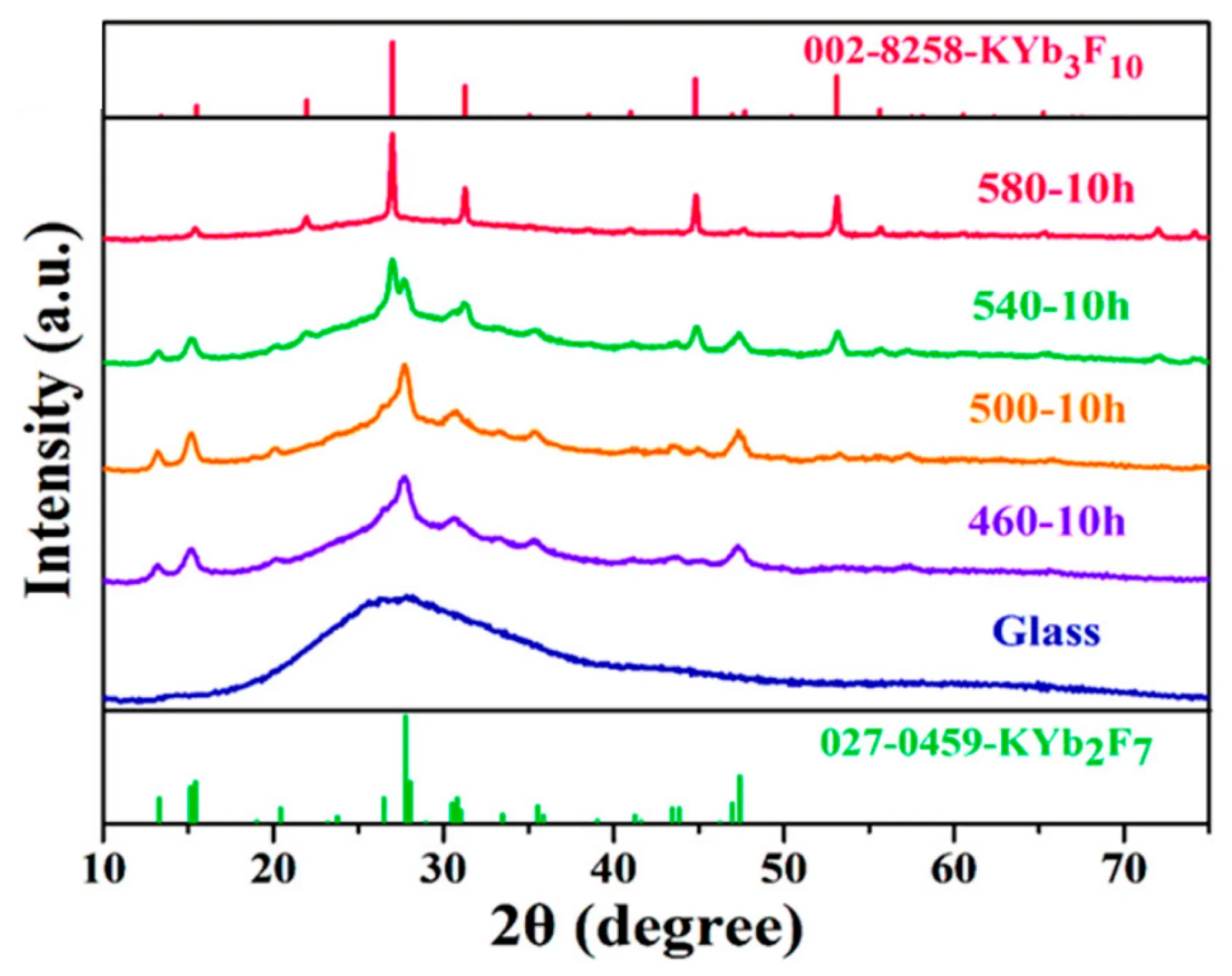
XRD patterns of the 1.0 mol% Yb^3+^/0.2 mol% Er^3+^-codoped 40Si–20B precursor glass and nanocrystallized glass-ceramics heat-treated at 460–580 °C for 10 h, showing the temperature-dependent evolution from KYb_2_F_7_ to KYb_3_F_10_. Adapted from Ref. [75] under the Creative Commons Attribution 4.0 International License (CC BY 4.0). Original Figure 2c of Ref. [75] was reformatted for presentation, and its panel designation was omitted; the underlying diffraction data were not altered.

The nominal 40SiO_2_–20B_2_O_3_–20KF–20ZnF_2_ composition, denoted as 40Si–20B, exhibited a particularly clear temperature-dependent phase evolution. As shown in Figure 18, the precursor glass displayed only a broad amorphous-scattering feature. After heat treatment at 460 and 500 °C for 10 h, the diffraction peaks were assigned predominantly to KYb_2_F_7_. Additional KYb_3_F_10_ reflections appeared after treatment at 540 °C, whereas KYb_3_F_10_ became the dominant detectable phase after treatment at 580 °C [75]. Thus, increasing the treatment temperature changed not only the extent of crystallization but also the identity of the Yb-containing fluoride phase.

Transmission electron microscopy of the sample treated at 580 °C showed nanocrystals approximately 5–30 nm in diameter that were dispersed within the residual glass matrix [75]. The reported lattice-fringe spacing of approximately 0.204 nm was assigned to the (440) plane of KYb_3_F_10_, providing microscopic support for the XRD phase assignment.

These results illustrate a dopant-associated crystallization pathway in which Yb^3+^ participates directly in the precipitated fluoride phases. Nevertheless, the simultaneous detection of a Yb-containing crystalline phase and enhanced luminescence does not establish the fraction of the total Yb^3+^ population incorporated into the nanocrystals. Quantitative phase analysis, spatially resolved compositional mapping, and site-selective spectroscopy would still be required to determine the partitioning of Yb^3+^ and Er^3+^ between the nanocrystals and the residual glass.

#### 6.2.3. Spectroscopic Consequences of Network Modification and Nanocrystallization

**Figure 19:**
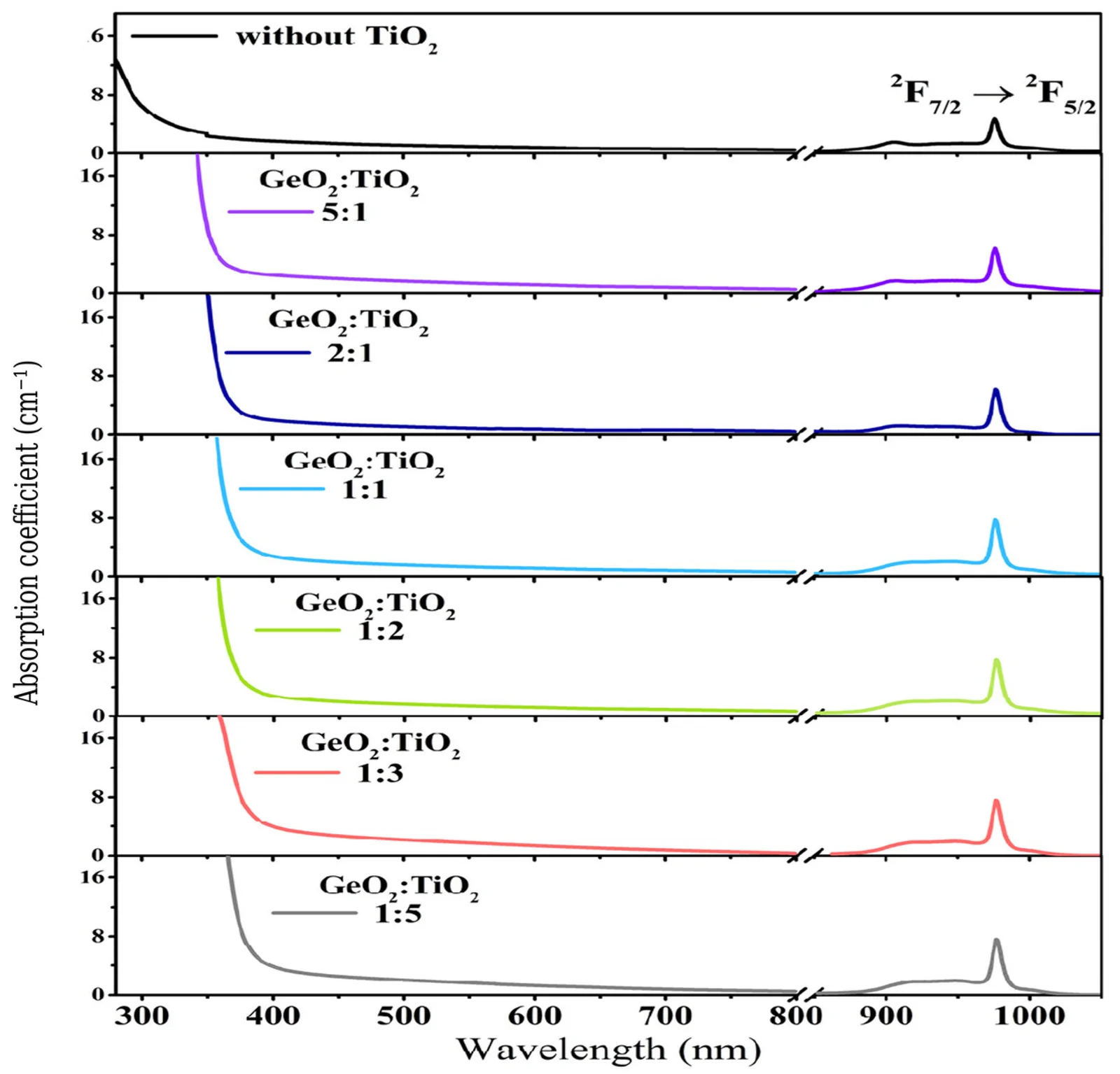
Absorption spectra of Yb^3+^-doped titanate–germanate glasses with different GeO_2_/TiO_2_ molar ratios, including the characteristic Yb^3+ 2^F_7/2_ → ^2^F_5/2_ absorption near 976 nm. Adapted from Ref. [76] under the Creative Commons Attribution 4.0 International License (CC BY 4.0); original Figure 3a was reformatted for presentation, and its panel designation was omitted; the underlying spectral data were not altered.

A complementary view of the sensitivity of Yb^3+^ to the surrounding glass network is provided by the xTiO_2_–(60−x)GeO_2_–30BaO–9.5Ga_2_O_3_–0.5Yb_2_O_3_ system, where x ranges from 0 to 50 mol% [76]. XRD confirmed that all investigated compositions remained amorphous. Figure 19 shows the characteristic Yb^3+^ absorption associated with the ^2^F_7/2_ → ^2^F_5/2_ transition. A resolved feature near 976 nm is observed, while the band shape and spectral distribution vary with the GeO_2_/TiO_2_ ratio.

The composition-dependent absorption profiles indicate that modification of the titanate–germanate network changes the distribution of local ligand fields and Stark components experienced by Yb^3+^. However, because all these samples remain XRD-amorphous, the spectral changes should not be interpreted as evidence of Yb^3+^ incorporation into a crystalline phase. Absorption spectroscopy is sensitive to the average Yb^3+^ environment, but it is not intrinsically phase-selective. Claims of Yb^3+^ partitioning into nanocrystals therefore require complementary diffraction, microscopy, lifetime, or site-selective spectroscopic evidence.

The spectroscopic consequences of controlled nanocrystallization can be evaluated more directly in the Yb^3+^/Er^3+^-codoped 40Si–20B fluoro-borosilicate system discussed above. Under 980 nm excitation, the precursor glass exhibits only weak upconversion emission, whereas the heat-treated samples show distinct Er^3+^ emission bands near 522, 540, and 650 nm, corresponding to the ^2^H_11/2_ → ^4^I_15/2_, ^4^S_3/2_ → ^4^I_15/2_, and ^4^F_9/2_ → ^4^I_15/2_ transitions, respectively [75].

**Figure 20:**
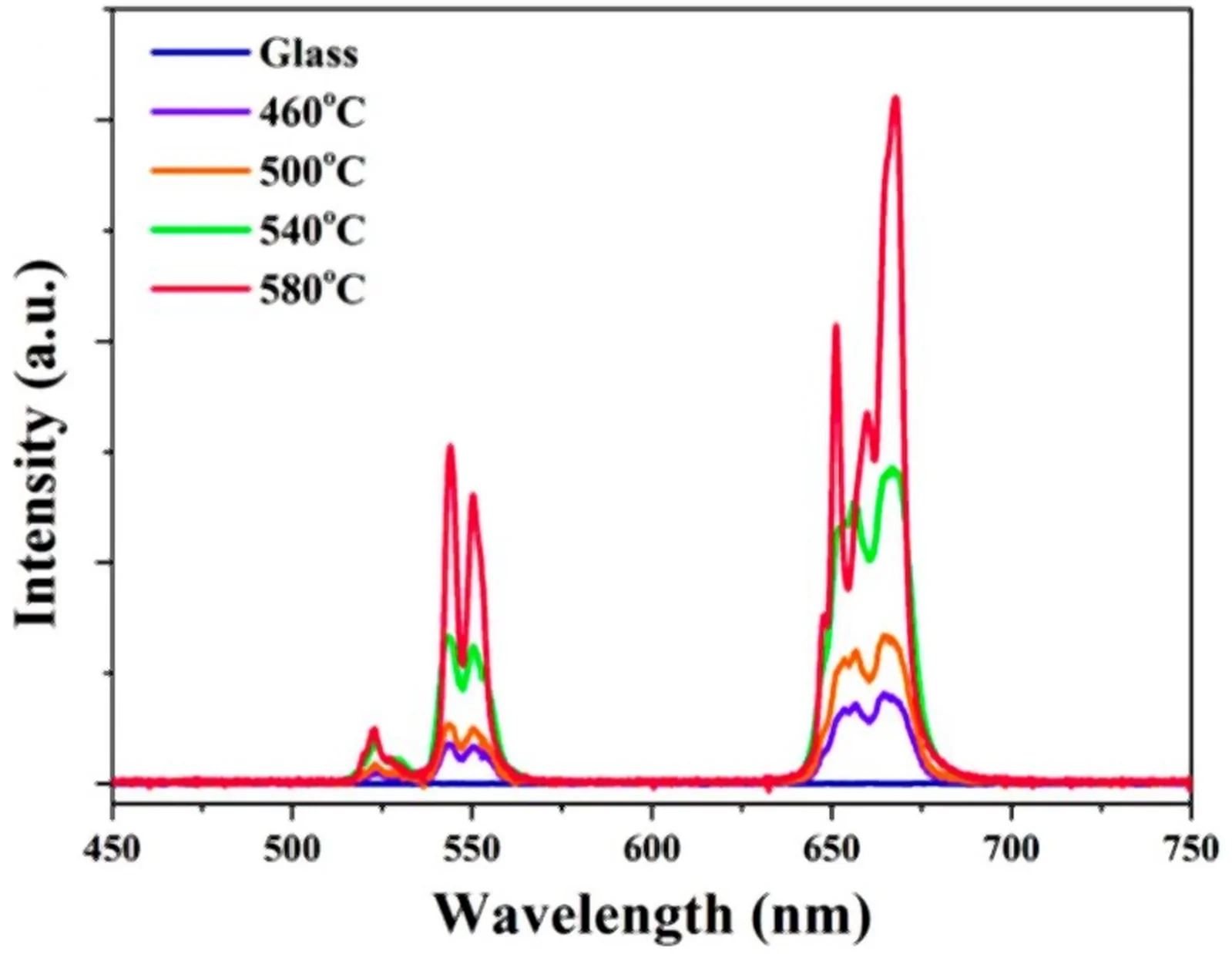
Upconversion emission spectra of the 1.0 mol% Yb^3+^/0.2 mol% Er^3+^-codoped 40Si–20B precursor glass and nanocrystallized glass-ceramics heat-treated at 460–580 °C for 10 h under 980 nm excitation. Adapted from Ref. [75] under the Creative Commons Attribution 4.0 International License (CC BY 4.0). Original Figure 3a was reformatted for presentation, and its panel designation was omitted; the underlying emission data were not altered.

As shown in Figure 20, the upconversion-emission intensity increases as the heat-treatment temperature rises from 460 to 580 °C. This enhancement occurs concurrently with the phase evolution from KYb_2_F_7_ to KYb_3_F_10_ identified by XRD. The low-phonon-energy fluoride environment reduces multiphonon relaxation and facilitates Yb^3+^-to-Er^3+^ energy transfer, providing a chemically plausible explanation for the enhanced green and red emissions.

The combined XRD and luminescence results establish a correlation between rare-earth-fluoride nanocrystallization and upconversion enhancement. Nevertheless, emission intensity is also affected by pump absorption, sample thickness, scattering, crystalline fraction, and the spatial distribution of the activator. Consequently, the stronger emission should not, by itself, be treated as a quantitative measurement of the fraction of Er^3+^ or Yb^3+^ incorporated into the crystalline phase.

#### 6.2.4. Yb^3+^-Modified ZnAl_2_O_4_ Nanocrystals

In 58SiO_2_-16[(1 − *x*%)Al_2_O_3_ + *x*%Yb_2_O_3_]-15ZnO-7Li_2_O-4TiO_2_-0.2Sb_2_O_3_ glasses, ZnAl_2_O_4_ nanocrystals were already detectable in the Yb-free composition after quenching, and heat treatment at 720 °C further developed the phase [102]. AsYb_2_O_3_ replaced Al_2_O_3_, Yb^3+^ entered the spinel-related nanocrystals, and the reported symmetry changed from $Fd\bar{3}m$ toward $F\bar{4}3m$.

This system is a counterexample to a simple nucleating-agent narrative: crystallization occurs without Yb^3+^, while Yb substitution modifies the structure of an existing crystallization pathway. The relevant role of Yb^3+^ is therefore phase modification and site substitution rather than the initiation of nucleation. A composition series measuring nucleation density and phase fraction would be needed to determine whether Yb also changes the rate.

### 6.3. What Promotes Heterogeneous Nucleation?

A constituent can promote nucleation by creating a compositionally distinct region, lowering the crystal–glass interfacial energy, increasing the thermodynamic driving force for a particular phase, or changing mobility. These mechanisms must be distinguished from merely observing that a phase appears after the constituent is added. In a multicomponent glass, the effective nucleating species may be a cluster or separated nanophase rather than the nominal oxide itself.

The SiO_2_-Li_2_O-K_2_O-ZnO-P_2_O_5_ series provides a useful control [103]. Increasing P_2_O_5_ changes the balance between surface and volume crystallization and produces phosphate-rich precursors that favor fine-grained lithium disilicate microstructures. The effect is associated with glass-network reorganization, phase separation, and interfacial energetics.

For Yb^3+^ systems, the available studies collectively suggest three recurring routes: (i) Yb/F-rich clustering before a Yb-bearing fluoride phase; (ii) Yb^3+^ substitution into a phase that already nucleates in the host; and (iii) Yb-induced network changes that alter viscosity or phase competition without placing most Yb^3+^ in the crystals. Distinguishing these routes requires in situ thermal/diffraction measurements, TEM, Raman spectroscopy, and element-specific local-structure probes [2,3,4,5,6,7,8,10,11,12,13,94,97,98,99].

### 6.4. Additional Mechanistic Evidence from Recent Yb^3+^-Containing Glass-Ceramics

Several mechanistic studies beyond the core case studies sharpen the interpretation of Yb-containing systems. Lin et al. showed that amorphous phase separation can predetermine fluoride-rich regions that later crystallize, while Bhattacharyya et al. and Bocker et al. demonstrated that growth may become self-limited or interface-controlled when crystallization changes the composition and viscosity of the surrounding glass [37,38,39]. Zhao et al. connected fluoride-rich domains to nanocrystal formation using solid-state NMR, STEM-EDS, and molecular-dynamics simulation [40]. These studies support phase separation as a plausible precursor, but they also show that phase-separated domains should not be equated with crystals unless diffraction or lattice imaging confirms the long-range order.

Ali et al. found that enhanced Er^3+^/Yb^3+^ upconversion after CaF_2_ precipitation was associated with increased structural ordering and changed local symmetry rather than requiring complete migration of the rare-earth ions into the fluoride crystals [41]. A related Yb^3+^/CaF_2_ germanotellurite study detected 8–10 nm nanocrystallites, but the amount and site occupancy of Yb^3+^ remained only partly constrained [42]. Collectively, these reports reinforce the need to combine optical spectra with direct chemical mapping or element-selective local-structure measurements.

Yb-only phosphate systems provide a useful control because sensitizer-to-activator transfer is absent. Sen et al. showed that Al_2_O_3_/Y_2_O_3_ additions strengthen the phosphate network, slightly slows crystallization, and favor surface crystallization, while precipitation of a Yb-containing phosphate phase changes the ^2^F_5/2_ lifetime and emission bandwidth [43]. Hongisto et al. obtained transparent NaYbP_2_O_7_-containing glass-ceramics only in selected NaF-free compositions and showed that NaF redirects bulk crystallization toward surface crystallization [44]. Their later composition study demonstrated that Al_2_O_3_, TiO_2_, or ZnO shifts the phase assemblage toward Na_5_P_3_O_10_ and suppresses Yb-containing phosphate phases [49]. These results indicate that network connectivity and modifier/intermediate balance can dominate over Yb concentration in determining phase selection.

Optical-refrigeration studies add quantitative criteria that are often absent from upconversion reports. de Lima Filho et al. found that the cooling figure of merit depends non-monotonically on the degree of crystallization [45]. Fiber and bulk studies subsequently linked performance to photoluminescence quantum efficiency, background absorption, OH^−^ content, scattering, temperature, and Yb^3+^ concentration [46,47,48]. These reports are particularly valuable because they measure absolute or near-absolute optical quantities; however, crystalline fraction and Yb partition coefficients should be linked more systematically to the measured cooling response.

Recent fluorosilicate and oxyfluoroborosilicate studies show that Yb^3+^ can be an active phase-forming constituent. Jamalaiah reported CaF_2_-containing Yb^3+^ glass-ceramics with useful 981 nm emission [50], while Fang et al. and Li et al. demonstrated controllable KYb_3_F_10_ precipitation and enhanced activator emission [51,53]. Long et al. used phase-separated fluoride-rich regions to alter activator distribution before crystallization [52]. The competitive formation of NaYbF_4_ and Na_3_ScF_6_ further shows that the Na/F ratio and competing-cation chemistry determine whether Yb enters the selected fluoride phase [60]. Xiang et al. extended this strategy by using B_2_O_3_ to lower the processing temperature and regulate KZnF_3_/KYb_3_F_10_ nanocrystallization [61]. These studies provide stronger evidence for Yb-induced phase selection than studies based only on DSC shifts, although such claims still require matched Yb-free controls and direct compositional mapping.

Ba_4_Y_3_F_17_, Ba_3_Gd_2_F_12_, ZnAl_2_O_4_, YOF, and GdOF glass-ceramics demonstrate that improved thermometric response can arise through lower phonon energy, changed site symmetry, crystallization-enhanced lifetime, or modified thermal coupling [54,55,56,57,58]. These studies report useful phase assignments and sensitivities, but application-level comparison remains difficult because excitation density, sample thickness, crystalline fraction, thermal cycling, and absolute quantum yield are not standardized. Pump-power-dependent measurements are especially important because the apparent fluorescence-intensity ratio and sensitivity may change when the upconversion pathway approaches saturation [57].

Finally, Blanc et al. demonstrated that nanoparticle composition can evolve before easily detectable crystallization [59]. This observation explains why early amorphous segregation may change the Yb^3+^ environment without producing sharp XRD peaks and provides a mechanistic link between glass-network heterogeneity and later phase selection. Taken together, the additional literature strengthens three conclusions: pre-nucleation segregation must be distinguished from crystallization; Yb^3+^ partitioning requires direct evidence; and optical enhancement should be evaluated together with crystalline fraction, transparency, lifetime, and absolute efficiency.

## 7. Composition Dependence: When Does Yb_2_O_3_ Promote or Suppress Crystallization?

Claims about the effect of Yb_2_O_3_ are often based on different base compositions, replacement schemes, and thermal protocols. A lower T_p_ in one glass and an amorphous XRD pattern in another are not contradictory unless the host network and phase field are comparable. The strongest evidence comes from a composition series in which Yb_2_O_3_ replaces a specified component while all other variables are fixed.

### 7.1. Yb-Free Barium–Zinc Borosilicate Kinetic Baseline

The crystallization kinetics of a barium–zinc borosilicate glass with the molar composition 40BaO-20ZnO-30B_2_O_3_-10SiO_2_ were examined using the Kissinger, Ozawa, and Augis–Bennett formalisms under non-isothermal conditions to determine the activation energy (E_c_) and Avrami exponent (*n*) [10,11,12,13,70]. These non-isothermal approaches are commonly employed to elucidate crystallization mechanisms, distinguish between surface- and volume-dominated crystallization, and assess the relative crystallizability of different glass compositions [10,11,12,13].

For the first exothermic event, the local activation energy *E_c_*(*χ*) fell from ~700 to ~500 kJ/mol as the crystallized fraction (χ) increased from 0.1 to 0.9. By contrast, the second exothermic peak exhibited a slight increase in *E_c_*(*χ*), from ~490 to ~570 kJ/mol, over the same χ range. The corresponding Avrami exponent *n*(χ) rose from ~1 to ~1.4 for the first peak, while it declined from ~1.7 to ~1.4 for the second peak. SEM observations of sintered samples revealed that crystallization begins at the particle surfaces, with lamellar crystallites growing inward at elevated temperatures, accompanied by quasi-spherical nano-sized crystallites. The dependence of the local activation energy on the crystallized fraction suggests that the overall process involves multiple kinetic steps during sintering and crystallization.

### 7.2. Yb_2_O_3_-Doped Barium Titanium Borate Glasses

The composition dependence of the parent-glass structure can be illustrated using the open-access Yb_2_O^3^-doped barium titanium borate system reported by Marzouk et al. [77]. The investigated compositions were 54.250B_2_O_3_–8.320TiO_2_–37.430BaO for the undoped glass and related compositions containing 0.258, 0.769, and 1.275 mol% Yb_2_O_3_.

**Figure 21:**
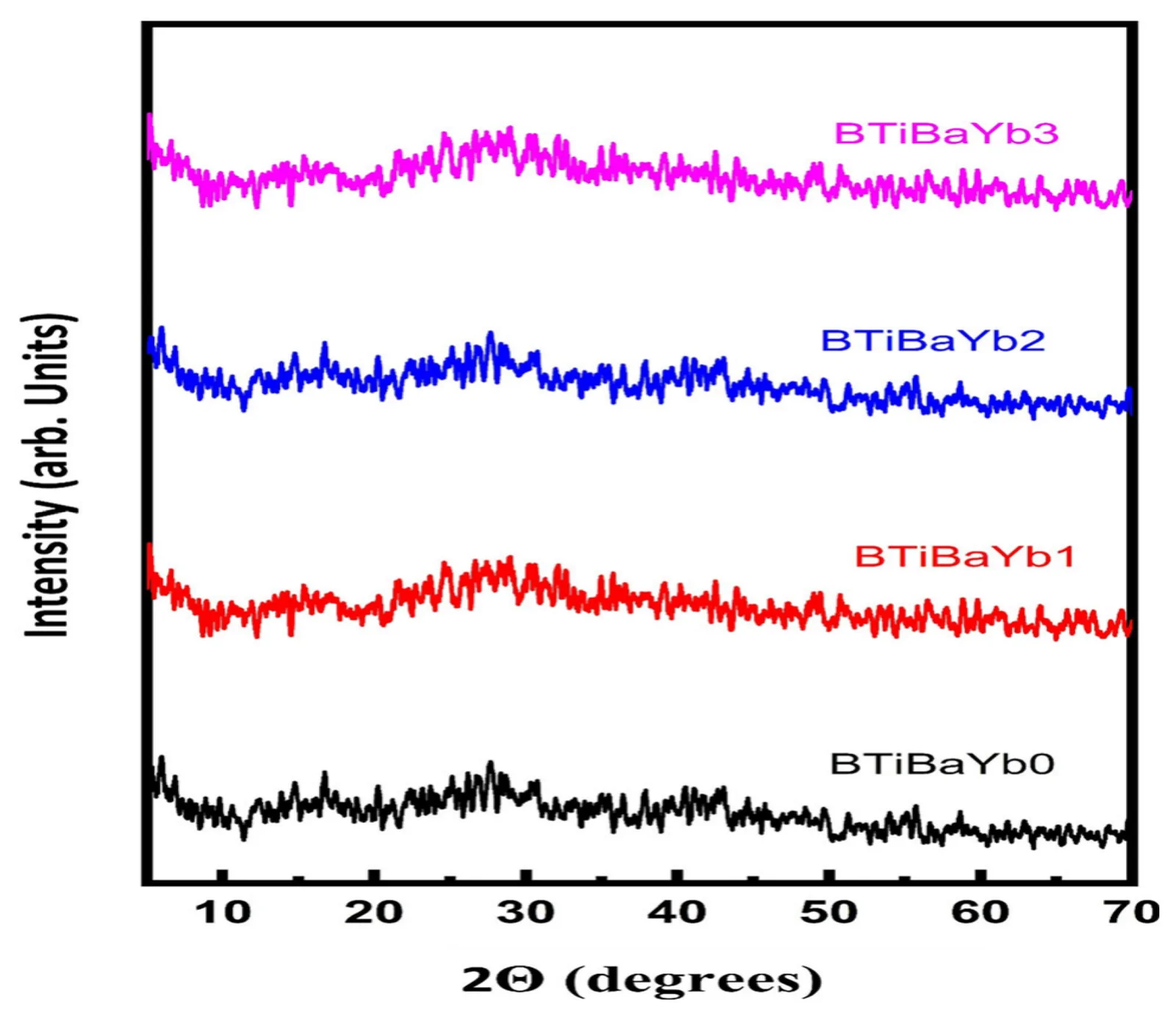
XRD spectra of Yb^3+^-doped barium titanium borate glasses containing 0–1.275 mol% Yb_2_O_3_; the broad amorphous-scattering features confirm the absence of detectable long-range crystalline order. Reproduced from Ref. [77] under the Creative Commons Attribution 4.0 International License (CC BY 4.0).

The XRD patterns shown in Figure 21 contain broad amorphous-scattering features without sharp Bragg reflections, confirming that all four as-prepared samples remained non-crystalline. The structural and physical trends discussed for this series therefore describe the modification of the parent-glass network rather than the development of a glass-ceramic phase.

**Figure 22:**
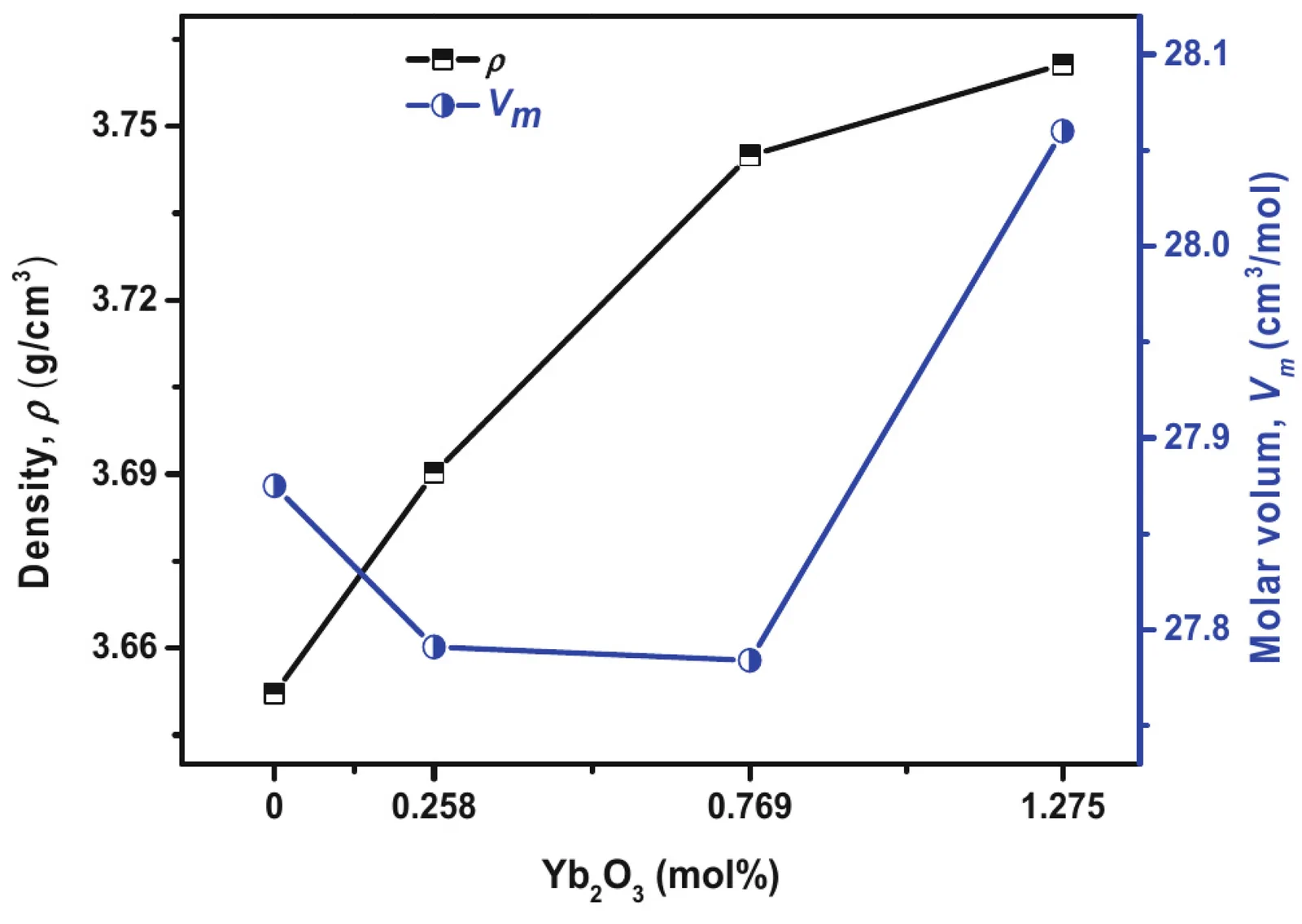
Density and molar volume of Yb^3+^-doped barium titanium borate glasses as functions of Yb_2_O_3_ content. Reproduced from Ref. [77] under the Creative Commons Attribution 4.0 International License (CC BY 4.0).

As shown in Figure 22, the measured density increased from 3.652 to 3.761 g cm^−3^ as the Yb_2_O_3_ content increased. The molar volume changed non-monotonically: it initially decreased from 27.875 to 27.784 cm^3^ mol^−1^ and subsequently increased to approximately 28.06 cm^3^ mol^−1^ at the highest Yb_2_O_3_ concentration [77]. Consequently, the density increase should not be interpreted simply as uniform network compaction; both the larger molar mass of Yb_2_O_3_ and composition-dependent changes in network packing contribute to the observed behavior.

Raman and FTIR measurements showed that Yb_2_O_3_ addition modified the distributions of BO_3_ and BO_4_ structural units. A characteristic broad Yb^3+^ absorption band near 977 nm, assigned to the ^2^F_7/2_ → ^2^F_5/2_ transition, became more intense with increasing Yb_2_O_3_ content. Over the same composition range, the optical band gap decreased from approximately 3.198 to 3.115 eV, whereas the calculated linear refractive index increased from approximately 2.346 to 2.367 [77].

These results confirm that Yb_2_O_3_ acts as an optically and structurally active component even when no crystalline phase is detected. However, the amorphous XRD patterns do not establish whether Yb_2_O_3_ promotes or suppresses subsequent crystallization. Resolving that question would require a matched heat-treatment series supported by DSC, quantitative XRD, and electron-microscopy measurements. The present results should therefore be interpreted as evidence for the composition-dependent modification of the precursor-glass network rather than direct evidence of Yb-induced nucleation.

## 8. Multiphase Crystallization and Phase Competition

### 8.1. Phosphate–Fluoride Glass

Yb^3+^-doped phosphate glasses with compositions (100 − x)(0.75NaPO_3_-0.25CaF_2_)-xYb_2_O_3_, with x = 3.0, were synthesized by a standard melt-quenching technique followed by a two-step thermal treatment [68]. The glass was first heat-treated at 320 °C, above the T_g_ (glass-transition temperature), and then heated at temperatures between 400 and 440 °C, corresponding to the crystallization range [68]. This system is useful for examining multiphase crystallization in phosphate–fluoride glasses containing Yb^3+^ [3,10,11,12,13,68].

**Figure 23:**
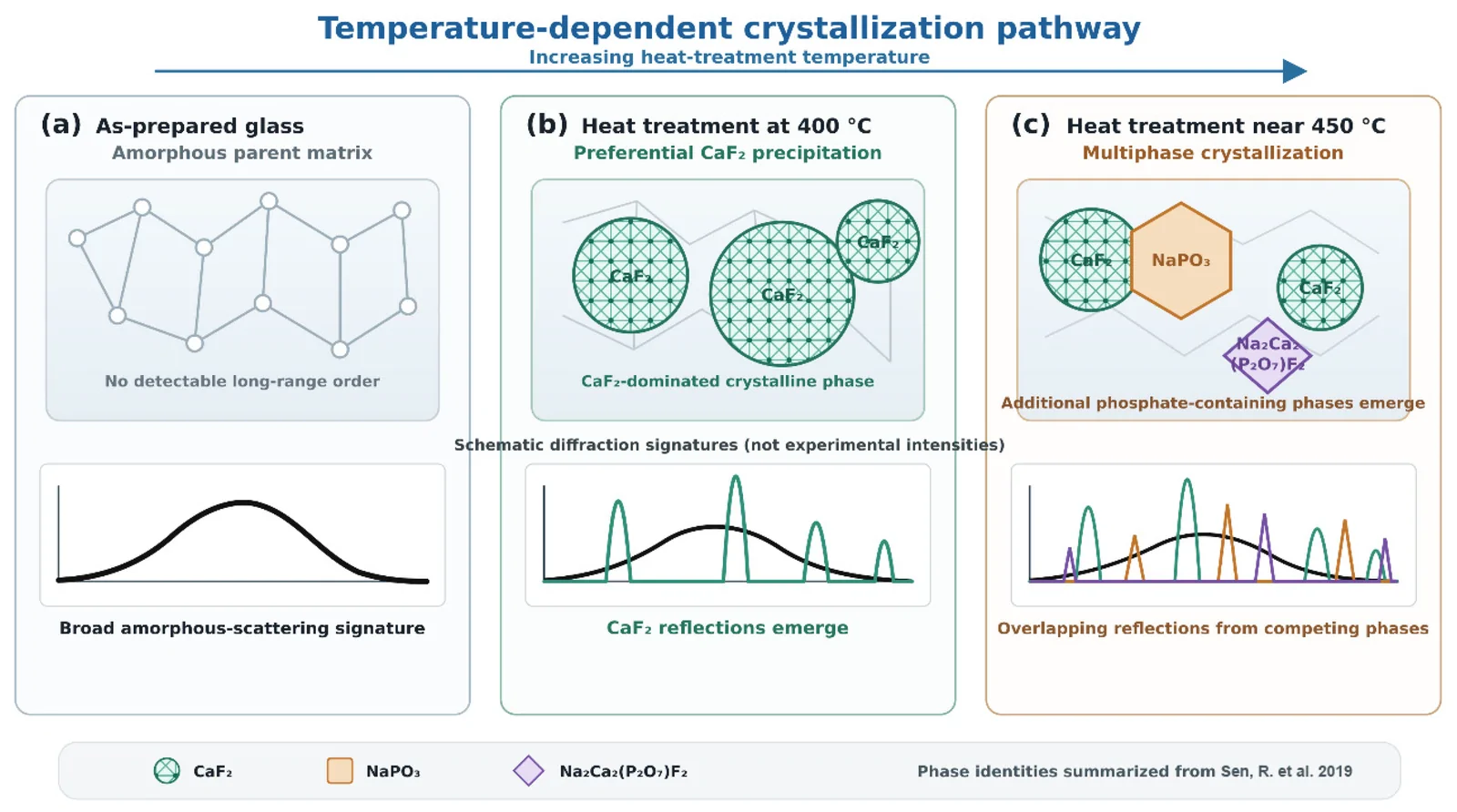
Author-generated schematic of the temperature-dependent crystallization pathway in Yb^3+^-doped phosphate–fluoride glass. (**a**) The as-prepared glass exhibits an amorphous structure; (**b**) CaF_2_ precipitates preferentially after heat treatment at 400 °C; and (**c**) additional NaPO_3_ and Na_2_Ca_2_(P_2_O_7_)F_2_ phases emerge near 450 °C, resulting in multiphase crystallization. The lower traces are qualitative diffraction signatures rather than experimental XRD patterns. The phase evolution is summarized based on Refs. [43,68].

The temperature-dependent phase evolution reported for the Yb^3+^-doped phosphate–fluoride glass is summarized schematically in Figure 23. Experimental XRD results reported in Ref. [68] showed that the as-prepared glass exhibited broad amorphous-scattering features without detectable sharp Bragg reflections. Heat treatment at 400 °C, close to the onset crystallization temperature (T_x_), resulted in the preferential precipitation of CaF_2_. When the heat-treatment temperature was increased to approximately 450 °C, near the reported crystallization-peak temperature (T_p_ = 452 °C), additional reflections assigned to NaPO_3_ and Na_2_Ca_2_(P_2_O_7_)F_2_ appeared, indicating multiphase crystallization [68]. A similar crystallization sequence has been reported for Er^3+^-doped glasses in the same system, in which CaF_2_ precipitated before the phosphate-containing phases [104]. SEM observations further supported the formation of crystalline domains. The lower traces in Figure 23 are qualitative diffraction signatures intended to illustrate the reported phase evolution and should not be interpreted as experimental XRD patterns.

SEM observations indicate that crystallization occurs throughout the glass volume when heat treatment is performed near *T_x_*, and the resulting crystals are approximately spherical. Elemental mapping identifies these crystals as CaF_2_, consistent with the XRD analysis. Increasing the heat-treatment temperature increases the number of crystals while reducing their average size. Yb^3+^ remains broadly distributed throughout the material, suggesting that part of the Yb^3+^ population is incorporated into the CaF_2_ crystals while the remainder stays in the residual glass matrix. Phosphate-rich crystals were not observed in SEM images of the sample treated at 450 °C, although they were detected by XRD, suggesting preferential surface crystallization. The emission spectra further indicate that CaF_2_ precipitation has little effect on the Yb^3+^ near-infrared ^2^F_5/2_ → ^2^F_7/2_ emission, whereas formation of NaPO_3_ and Na_2_Ca_2_(P_2_O_7_)F_2_ substantially reduces the emission intensity.

### 8.2. Ytterbium Aluminosilicate Glass

The 15Yb_2_O_3_-25Al_2_O_3_-60SiO_2_ system represents the opposite composition limit: Yb_2_O_3_ is a major constituent rather than a dilute additive [69,85,97,105,106,107]. The as-prepared bulk glass combines a high T_g_ (847 °C), low room-temperature thermal conductivity, and substantial mechanical strength. At 1200 °C, it crystallizes into Yb_3_Al_5_O_12_, Yb_2_Si_2_O_7_, and Al_6_Si_2_O_13_.

The formation of two Yb-bearing phases demonstrates that Yb^3+^ can be incorporated directly into the crystalline assemblage when its concentration is high. Phase selection is governed by the Yb_2_O_3_–Al_2_O_3_–SiO_2_ stoichiometry and heat treatment, not by a generic “Yb nucleating-agent” effect. Competing phases also mean that a single optical or mechanical trend cannot be assigned to one crystal without quantitative phase analysis.

Rare-earth aluminosilicate glasses are of interest for plasma-resistant components because rare-earth fluorides formed at exposed surfaces can have low volatility [69,105]. This application is relevant to the stability of the material but is secondary to the crystallization mechanism. The present review therefore retains only the composition–phase–property connection and removes the extended semiconductor-processing description.

The phosphate–fluoride and aluminosilicate cases define two ends of a design spectrum. At low Yb content, Yb^3+^ may partition only partly into an early fluoride nanophase; at high content, several Yb-rich oxide phases can consume most of the glass. Between these limits, controlling phase competition is more important than maximizing a nominal nucleation rate.

### 8.3. Integrated Roles of Yb^3+^ in Crystallization and Luminescence

**Figure 24:**
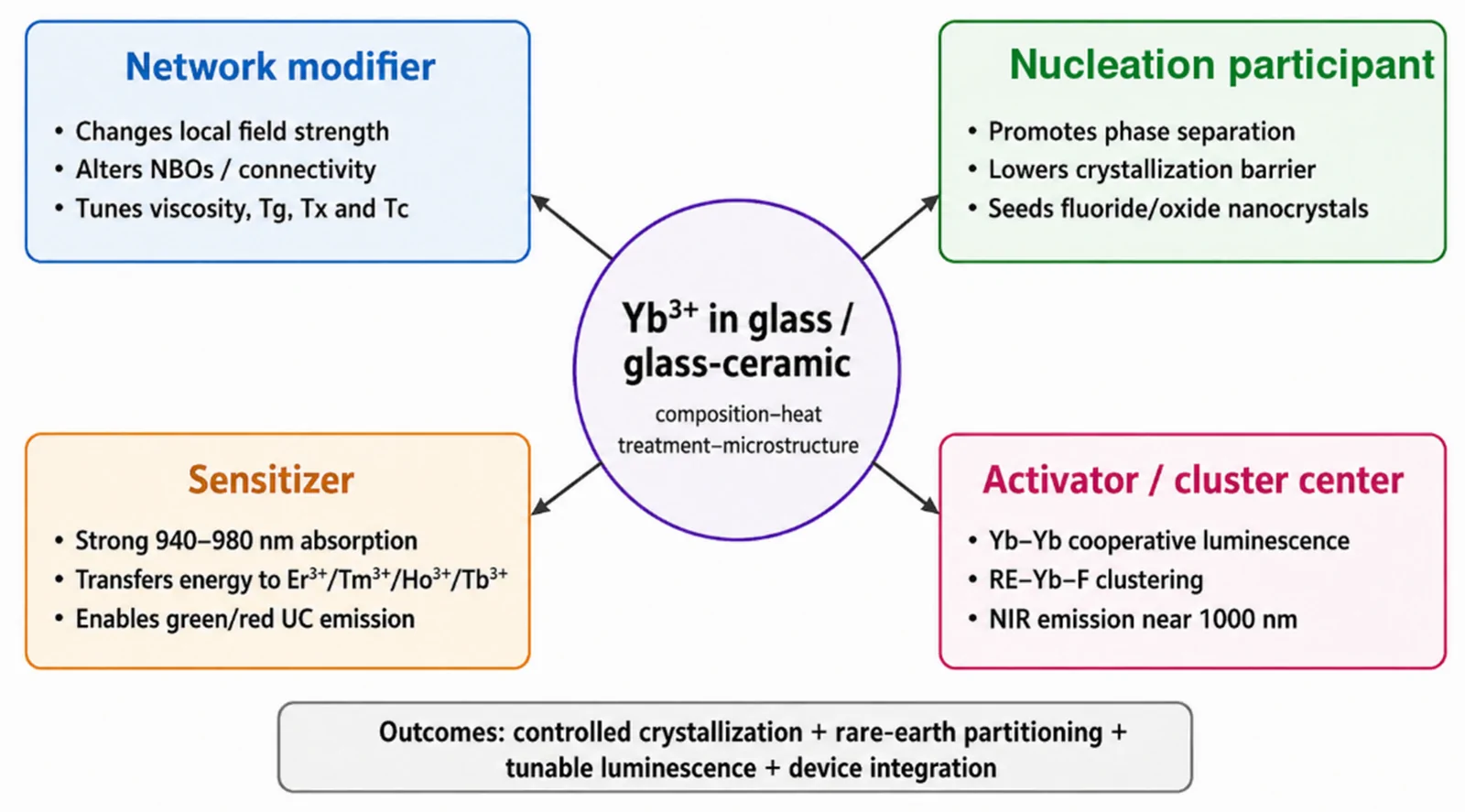
Schematic summary of the four major roles of Yb^3+^ in crystallization and luminescence of glasses and glass-ceramics: network modifier, participant in nucleation precursors, sensitizer, and activator/cluster center. The same Yb^3+^ component can therefore influence both phase formation and optical response, depending on glass composition, local coordination, clustering, and heat-treatment schedule.

The preceding examples demonstrate that Yb^3+^ cannot be assigned a single universal function in glasses and glass-ceramics. Depending on the parent-glass composition, Yb^3+^ concentration, local coordination environment, codopants, and heat-treatment schedule, it may act as a glass-network modifier, participate in nucleation precursors, serve as an optical sensitizer, or function as an emitting center and a component of Yb-rich clusters. These four composition-dependent roles and their relationships with crystallization and luminescence are summarized in Figure 24.

As a glass-network modifier, Yb^3+^ can alter the connectivity and packing of the parent-glass network. Its relatively high field strength and coordination requirements may change the population of non-bridging oxygen atoms, modify the association of fluoride or phosphate species, and influence viscosity, density, and the thermal stability window. However, the direction and magnitude of these changes depend on the host network. Yb_2_O_3_ may depolymerize one glass network while strengthening or stabilizing another; therefore, it should not be assumed to exert the same structural effect in silicate, phosphate, borate, and oxyfluoride glasses [43,44,49,65,87,88].

Yb^3+^ may also participate in the formation of nucleation precursors when it becomes enriched in modifier-rich, fluoride-rich, or phosphate-rich regions. If the local composition and coordination of these regions resemble those of the emerging crystalline phase, the effective crystal–glass interfacial barrier may be reduced, and nucleation may become more favorable. This behavior is particularly relevant when Yb^3+^ can enter CaF_2_-related solid solutions or form Yb-containing phases such as Pb_4_Yb_3_F_17_, Yb_3_Al_5_O_12_, or Yb_2_Si_2_O_7_. Conversely, when Yb^3+^ stabilizes the residual glass, increases configurational disorder, or is incompatible with the initially precipitating phase, it may slow crystallization or redirect the process toward competing crystalline phases. Yb^3+^ should therefore be described as a composition-dependent participant in nucleation rather than as a universal nucleating agent [37,38,39,40,41,42,43,44,50,51,52,53,59,60,61,64,65,66,67,68,69,102].

The third role is optical sensitization. Yb^3+^ efficiently absorbs near-infrared radiation in the approximately 900–1000 nm range through the ^2^F_7/2_ → ^2^F_5/2_ transition and can subsequently transfer excitation energy to neighboring activator ions such as Er^3+^, Ho^3+^, or Tb^3+^. Controlled incorporation of Yb^3+^ and the activator into a low-phonon-energy crystalline phase may reduce non-radiative relaxation and enhance upconversion emission. Nevertheless, stronger steady-state emission does not by itself prove that Yb^3+^ has entered the nanocrystals, because heat treatment can also change pump absorption, scattering, activator–sensitizer distances, and the composition of the residual glass [18,19,20,41,51,52,53,54,55,56,57,58,62,63,64,65,66,67].

Finally, Yb^3+^ can itself act as an optically active center through the ^2^F_5/2_ → ^2^F_7/2_ near-infrared emission. At higher local concentrations, neighboring Yb^3+^ ions may form pairs or clusters that support cooperative luminescence. Such aggregation can be beneficial when cooperative emission is the desired function, but it may also enhance energy migration, cross-relaxation, and concentration quenching. The optimal Yb^3+^ concentration is therefore determined by a balance among pump absorption, rare-earth partitioning, inter-ionic distance, multiphonon relaxation, and clustering [18,19,20,50,51,52,53,87,88,89,90].

The four roles shown in Figure 24 are not independent. Network modification can create compositionally distinct precursor regions; these regions may subsequently nucleate Yb-containing nanocrystals; crystallization then redistributes Yb^3+^ and codoped activators between the crystalline phases and the residual glass; and this redistribution changes both energy-transfer efficiency and concentration-quenching behavior. Accordingly, composition and heat treatment must be optimized jointly. A reliable interpretation requires thermal analysis, phase identification, microscopy, local-structure measurements, and quantitative optical characterization to follow the same Yb^3+^ population from the parent glass to the final glass-ceramic.

## 9. Conclusions

The surveyed evidence supports a conditional, rather than universal, picture of Yb^3+^-controlled crystallization. Yb^3+^ can promote fluoride-rich precursor formation, enter CaF_2_-related or Pb_4_Yb_3_F_17_ nanocrystals, substitute into spinel- or oxyapatite-related lattices, or remain largely in the residual glass while modifying its network and viscosity. Which role dominates is determined by the parent composition, Yb^3+^ concentration, charge-compensation chemistry, and thermal schedule.

### 9.1. Practical Design Guidelines

First, define the target phase and the intended Yb^3+^ site before selecting a heat treatment. A fluoride nanophase designed to host Yb^3+^/Er^3+^ requires different precursor chemistry from an oxide glass intended to crystallize Yb_3_Al_5_O_12_. Phase diagrams and chemically plausible oxidation states should constrain the proposed pathway.

Second, separate nucleation from growth experimentally. Use multiple heating rates to estimate apparent kinetics, then arrest samples across each thermal event and correlate DSC/DTA with XRD, TEM, and Raman measurements. An Avrami exponent or a ΔT value should be treated as supporting evidence rather than a stand-alone mechanism.

Third, optimize a multi-objective response. Crystal fraction and luminescence should be evaluated together with transmission, size distribution, and thermal stability. The best glass-ceramic is commonly obtained before the strongest XRD peak or longest anneal, because coarsening and secondary phases can reduce optical performance.

Fourth, determine where Yb^3+^ resides. Steady-state emission enhancement is not sufficient proof of incorporation. EXAFS/XANES, site-selective spectroscopy, lifetime components, elemental mapping, or another direct/quantitative method should be used to distinguish crystal-hosted Yb^3+^ from clusters and residual-glass sites [98,100,101].

Fifth, report negative and non-monotonic results. A composition in which Yb_2_O_3_ suppresses a phase, shifts the crystallization peak upward, or preserves an amorphous structure is mechanistically valuable. Such data reveal the boundary between nucleation promotion, network stabilization, and phase competition.

For codoped systems, optical optimization also requires control of Yb-Yb and Yb-Ln distances. Increasing Yb^3+^ can not only improve pump absorption but also strengthen clustering, cooperative luminescence, cross-relaxation, or concentration quenching. Composition and heat treatment must therefore be designed jointly.

A minimum comparative dataset should include the complete parent-glass composition, replacement basis, T_g_/T_x_/T_p_ at specified heating rates, nucleation and growth schedules, phase identity, crystalline fraction, image-based size distribution, Yb^3+^ partitioning, transmission, lifetime, absolute quantum efficiency, and stability after repeated thermal exposure.

### 9.2. Unresolved Questions and Reporting Priorities

The largest unresolved question is the identity of the earliest Yb-associated precursor. Fluoride-rich clusters, phosphate-rich domains, and modifier-related non-bridging-oxygen environments are frequently inferred but rarely observed before XRD-detectable crystals form. In situ total scattering, synchrotron X-ray absorption, small-angle scattering, and high-resolution microscopy on time-resolved samples could close this gap.

A second gap is comparability. Activation energies obtained by different non-isothermal methods are often compared without confirmation of identical transformed fractions or phase pathways. Community reporting of raw thermal curves, fitting intervals, uncertainty, and phase evolution would make cross-study comparisons more defensible.

A third gap is quantitative structure–optical correlation. Many studies report relative intensity enhancement but omit absorbed pump power, lifetime, quantum efficiency, and crystalline fraction. Without these quantities, it is difficult to determine whether crystallization improved intrinsic radiative efficiency or merely changed absorption and scattering.

Finally, environmental and manufacturing constraints should enter materials selection early. Lead- and cadmium-containing oxyfluoride systems offer clear mechanistic examples but present volatility and toxicity concerns. Lead-free compositions, scalable heat treatments, reproducible nanocrystal distributions, and long-term optical stability are necessary for translation.

In summary, Yb^3+^ is best viewed as a composition-dependent participant in a coupled glass-network, crystallization, and energy-transfer system. Treating it only as an optical dopant or universally as a nucleating agent obscures the central mechanism. Progress will come from measurements that follow the same Yb population from the parent glass, through the precursor stage, into the final crystal and residual matrix.

The revised framework turns the available case studies into testable design rules while making the limitations explicit. It also explains why nominally conflicting effects of Yb_2_O_3_ can coexist across glass families: the host network determines whether Yb^3+^ lowers an interfacial barrier, alters mobility, enters the product phase, or stabilizes the amorphous state.

## Figures and Tables

**Table 1:** Comparative crystallization mechanisms, structural evidence, optical performance, and reporting gaps in representative Yb^3+^-containing glass systems. QE: quantum efficiency; PG: precursor glass; GC: glass-ceramic; UC: upconversion; *n*: the Avrami exponent.

System/Reference	Thermal Schedule	Phase and Size	Kinetic Evidence/Parameters	Yb Role/Optical Response	Transparency, Thermal Stability, and Interpretation
45SiO_2_-15Al_2_O_3_-12Na_2_O-21BaF_2_-7YF_3_; Er^3+^/Yb^3+^ [62]	Controlled heat treatment after melt quenching	Cubic BaYF_5_; ~10 nm domains and ~60–65 nm particles	No E_a_ or crystalline fraction reported	Preferential RE incorporation is inferred from Stark splitting. Er^3+^ UC occurs at 523, 541, and 653 nm with an approximately twofold enhancement; lifetime and QE were not reported.	>80% transmittance was retained in the measured range, and stability against further crystallization was reported at the sensing temperature. The optical data support a lower-phonon environment, but direct Yb/Er partition coefficients and crystalline fraction are unavailable.
20Na_2_O-42ZnO-28P_2_O_5_-10B_2_O_3_; Yb^3+^/Ho^3+^/Tb^3+^ [63]	Annealed at 420 °C; crystallized at 600 °C for 4 h	NaZnPO_4_; numerical size not reported	T_g_ = 457 °C; T_x_ = 537 °C; T_p_ = 643 °C	Yb^3+^ acts mainly as a sensitizer; partitioning is inferred. UC bands occur at 414, 436, 489, 546, 586, 621, and 659 nm; lifetime and QE were not reported.	A transparent GC was obtained, but thermal stability was not quantified. Emission enhancement is clear, whereas crystalline fraction, lifetime, and absolute UC QE remain unreported.
SiO_2_-Al_2_O_3_-CaF_2_; Tb^3+^/Yb^3+^ [64]	660 °C for 2 h	CaF_2_ nanocrystals; TEM confirmation	E_a_ ≈ 498 kJ mol^−1^; *n* ≈ 1.01	TbF_3_/YbF_3_ lowers the crystallization temperature by ~42 °C. Yb-sensitized emission is phase-dependent; lifetime and QE were not consistently reported.	Transparency is retained when the crystals remain nanoscale, but thermal stability was not quantified. The *n* value is compatible with constrained growth, although morphology and data from multiple heating rates are needed for a firm mechanism assignment.
NaF-CaF_2_-Al_2_O_3_-SiO_2_; variable Yb_2_O_3_ [65]	Heat treatment near the crystallization range	CaF_2_/Ca_0.8_Yb_0.2_F_2.2_; ~10–20 nm	Apparent E_a_ decreases with increasing Yb content	Yb^3+^ substitutes into a CaF_2_-related phase and produces a phase-dependent optical response; lifetime and QE were not consistently reported.	Transparency can be retained for the reported nanocrystal sizes. This is one of the strongest cases for Yb-assisted fluoride crystallization, but the relationship among crystalline fraction, Yb occupancy, emission efficiency, and long-term stability requires quantitative evaluation.
SiO_2_-B_2_O_3_-PbO-CdO-PbF_2_-CdF_2_; Er^3+^/Yb^3+^ [66]	683–703 K for 3–30 h	Pb_4_Yb_3_F_17_-related nanocrystals; self-constrained growth	DTA/XRD time series; kinetically self-constrained model	Er-Yb-F-rich clusters act as precursors, with RE enrichment in fluoride regions. Yb NIR and Er^3+^ Stokes/anti-Stokes emissions are observed; lifetime and QE were not reported.	A stable nanocrystallization window was reported, although short-wavelength scattering increased. Clustering plausibly links phase separation to nucleation, but quantitative crystalline fraction, RE site occupancy, and direct pre-nucleation composition maps remain unavailable.
53SiO_2_-36Na_2_O-9Y_2_O_3_-2P_2_O_5_; Yb^3+^/Ho^3+^/Tb^3+^ [67]	670 °C/1 h + 770 °C/40 min	Na_3_YSi_2_O_7_; ~100–200 nm	E_a_ = 580–598 kJ mol^−1^; crystallization index ≈ 1	RE incorporation into Y sites is inferred from luminescence. Tb^3+^ green emission is enhanced by ~7 times; lifetime and QE were not reported.	Higher crystallinity increases emission but reduces transmittance. Thermal stability was described qualitatively, whereas absolute transmittance at the application wavelength, lifetime, QE, and crystalline fraction were not quantified.
97(0.75NaPO_3_-0.25CaF_2_)-3Yb_2_O_3_ [68]	320 °C, then 400–450 °C	CaF_2_ first, then NaPO_3_ and Na_2_Ca_2_(P_2_O_7_)F_2_	Sequential phase formation from XRD/SEM	Yb^3+^ appears distributed between the glass and CaF_2_-rich regions. NIR emission is retained after CaF_2_ formation but decreases when phosphate-rich phases appear; lifetime and QE were not reported.	Transparency and thermal stability were not quantitatively reported. XRD and SEM detect different crystal populations, and direct evidence for Yb distribution among the three phases is still required.
15Yb_2_O_3_-25Al_2_O_3_-60SiO_2_ [69]	Complete crystallization at 1200 °C	Yb_3_Al_5_O_12_, Yb_2_Si_2_O_7_, and Al_6_Si_2_O_13_	No kinetic parameters or size distribution reported	Yb is a major crystal-forming constituent; optical emission was not the main focus.	The material has high T_g_ and good thermal stability but was not designed as a transparent GC after complete crystallization. The optical consequences of the multiphase assemblage remain insufficiently characterized.
Barium–zinc borosilicate and related borate glasses; variable Yb_2_O_3_ [70,71,72,73,74]	Melt-quenched glasses; thermal and structural analyses over composition-dependent Yb_2_O_3_ contents	No crystalline phase identified in the reported Yb-doped glasses	The undoped borosilicate shows composition-dependent E_a_ and *n*; Yb-doped glasses show modest T_g_, T_c_, and ΔT changes	Yb_2_O_3_ modifies packing, thermal response, band gap, and refractive index; lifetime and QE are not applicable or were not reported.	The parent glasses remain XRD-amorphous. These data demonstrate network modification rather than a direct nucleating effect; controlled crystallization experiments are required before assigning Yb_2_O_3_ a crystallization-promoting role.
Phase-separated fluorosilicate and oxyfluoride glasses [37,38,39,40,41,42,59]	Heat treatment within the T_g_-T_p_ window; time- and temperature-dependent series	KZnF_3_/K_2_SiF_6_ and BaF_2_/CaF_2_-related nanophases; sub-10 to tens of nm	Amorphous phase separation, residual-glass depletion, and self-limited/interface-controlled growth	Local ordering and RE partitioning are system-dependent and only partly quantified. Optical enhancement can arise from changed local symmetry even without complete RE migration into the crystals.	Transparency depends on crystal size, fraction, and refractive-index mismatch. The strongest mechanistic evidence combines NMR, STEM/TEM, chemical mapping, and simulation; optical enhancement alone is insufficient to establish quantitative partitioning.
Yb^3+^-containing phosphate glasses and glass-ceramics [43,44,49]	Annealing near T_g_ and T_p_ across several modifier/intermediate compositions	NaYbP_2_O_7_, NaPO_3_, and Na_5_P_3_O_10_	Additives shift bulk crystallization toward surface crystallization and redirect phase selection	Yb^3+^ acts as an emitting ion and, in selected compositions, a crystal-forming constituent. The ^2^F_5/2_ lifetime and NIR emission bandwidth change after Yb-containing phase precipitation.	Transparency is retained only in selected compositions, and the crystallization window depends strongly on additives. Network connectivity can dominate over Yb concentration; direct Yb distribution and quantitative crystalline fraction remain limited.
Yb^3+^ glass-ceramics for optical refrigeration [45,46,47,48]	Controlled partial crystallization of bulk samples and fibers	Yb-containing fluoride nanocrystals in oxyfluoride matrices	Cooling performance is non-monotonic with crystallization; complete kinetic models are generally absent	Yb^3+^ is both the refrigerant ion and a possible participant in the precipitated phase. High or near-unity internal QE is reported for selected compositions, whereas background absorption is critical.	Transparency and cooling depend on nanocrystal size, fraction, scattering loss, OH^−^ content, and temperature. Phase fraction and Yb partition coefficients should be linked quantitatively to cooling efficiency and long-term thermal cycling.
CaF_2_, KYb_3_F_10_, and NaYbF_4_ systems [50,51,52,53,60,61]	Melt quenching followed by composition-dependent annealing or controlled self-crystallization	CaF_2_, KYb_3_F_10_, NaYbF_4_, KZnF_3_, and competing Na_3_ScF_6_	Dopant- and composition-controlled phase competition; phase separation may precede crystallization	Yb^3+^ is a sensitizer and, in KYb_3_F_10_/NaYbF_4_, an essential phase-forming cation. Strong NIR or UC emission is reported; an absolute UC QE of 1.44 ± 0.02% was obtained for one KYb_3_F_10_-based system [51].	Transparent samples are obtained only under selected crystallization conditions, and thermal testing generally covers limited windows. Claims of Yb-induced crystallization require matched Yb-free controls, direct partition maps, and long-term optical-stability data.
Thermometric fluoride and oxide glass-ceramics [54,55,56,57,58]	Melt quenching plus annealing or self-crystallization	Ba_4_Y_3_F_17_, Ba_3_Gd_2_F_12_, ZnAl_2_O_4_, YOF, and GdOF	Detailed crystallization kinetics are reported only for selected systems	Yb^3+^ serves mainly as a sensitizer and may also enter the crystalline phase. Enhanced UC and FIR thermometry are reported; lifetime enhancement is available in selected systems, but absolute QE is usually absent.	Transparent GCs and useful sensitivities are reported, but thermal-cycling protocols vary. Pump power density, sample thickness, crystalline fraction, cycling, and absolute QE require standardization.
(60 − x)SiO_2_–xB_2_O_3_–20KF–20ZnF_2_; 1.0Yb^3+^/0.2Er^3+^ [75]	460–580 °C for 10 h	KYb_2_F_7_ at lower temperatures; transition to KYb_3_F_10_ at higher temperatures; approximately 5–30 nm	Temperature-dependent XRD and TEM/HRTEM	Yb^3+^ is both sensitizer and phase-forming constituent; Er^3+^ upconversion bands near 522, 540, and 650 nm increase with heat-treatment temperature.	The system provides direct phase-evolution evidence, but crystalline fraction and rare-earth partition coefficients were not quantified.
Yb^3+^-doped titanate–germanate and barium titanium borate parent glasses [76,77]	Melt-quenched, as-prepared glasses; no post-crystallization schedule in the cited datasets	No crystalline phase detected by XRD	Composition-dependent absorption, density, molar volume, Raman/FTIR, and amorphous XRD	Yb^3+^ modifies the glass network and near-infrared absorption; crystalline partitioning cannot be inferred.	These datasets establish parent-glass modification rather than crystallization; matched heat-treatment series are required to test nucleation effects.

Notes: T_g_ is the glass-transition temperature; T_x_ is the onset crystallization temperature; T_p_ is the peak crystallization temperature measured by differential scanning calorimetry (DSC) or differential thermal analysis (DTA); and T_c_ is the crystallization temperature as defined in the cited source. ΔT is the empirical thermal-stability or crystallization window, generally calculated as T_x_ − T_g_. E_a_ is the apparent activation energy for crystallization, commonly obtained using non-isothermal Kissinger, Ozawa, Matusita, or related analyses. The Avrami exponent *n* gives model-dependent information on nucleation behavior and growth dimensionality.

## Data Availability

No new data were created or analyzed in this study. Data sharing is not applicable to this article.
